# Effect of maternal separation and transportation stress on the bovine upper respiratory tract microbiome and the immune response to resident opportunistic pathogens

**DOI:** 10.1186/s42523-021-00123-2

**Published:** 2021-09-19

**Authors:** Nilusha Malmuthuge, Angela Howell, Natasa Arsic, Tracy Prysliak, Jose Perez-Casal, Philip Griebel

**Affiliations:** 1 Vaccine & Infectious Disease Organization – International Vaccine Centre (VIDO-InterVac), University of Saskatchewan, Saskatoon, SK Canada; 2 Present Address: Agriculture and Agri-Food Canada, Lethbridge Research and Development Centre, 5403 1 Ave S, Lethbridge, AB T1J 4B1 Canada; 3 School of Public Health, University of Saskatchewan, Saskatoon, SK Canada

**Keywords:** Stress, Upper respiratory tract, Microbiome, Adrenergic receptors, Antibody response, Bovine

## Abstract

**Background:**

The bovine upper respiratory tract (URT) microbiome includes opportunistic pathogens that cause respiratory disease and stress associated with maternal separation and transportation contributes to the severity of this respiratory disease. Stress is known to alter the gut microbiome but little is known regarding the effect of stress on the URT microbiota. This study used six-month old suckling beef calves to investigate whether maternal separation (weaned), by itself or combined with transportation (weaned + transport), altered the URT microbiome and host immune responses to resident opportunistic pathogens.

**Results:**

Taxonomic and functional composition of the URT microbiome in suckling and weaned beef calves did not change significantly when serially sampled over a one-month period. Subtle temporal changes in the URT microbiome composition were observed in weaned + transport calves. Total bacterial density was lower (*p* < 0.05) on day 4 post-weaning in both the weaned and weaned + transport groups when compared to suckling calves. In addition, significant (*p* < 0.05) temporal changes in the density of the opportunistic pathogens, *M. haemolytica* and *P. multocida*, were observed independent of treatment but these changes did not correlate with significantly increased (*p* < 0.05) serum antibody responses to both of these bacteria in the weaned and weaned + transport groups. Serum antibody responses to *My. bovis*, another opportunistic pathogen, remained unchanged in all treatment groups. Weaning, by itself and in combination with transportation, also had significant (*p* < 0.05) short- (2 to 8 days post-weaning) and long-term (28 days post-weaning) effects on the expression of adrenergic receptor genes in blood leukocytes when compared to age-matched suckling beef calves.

**Conclusions:**

Maternal separation (weaning) and transportation has minor effects on the taxonomic and functional composition of the URT microbiome and temporal changes in the density of opportunistic pathogen residing in the URT did not correlate with significant changes in immune responses to these bacteria. Significant changes in adrenergic receptor expression in blood leukocytes following weaning, with or without transportation, suggests altered neuroimmune regulation should be further investigated as a mechanism by which stress can alter host-microbiome interactions for some opportunistic respiratory pathogens that reside in the URT.

**Supplementary Information:**

The online version contains supplementary material available at 10.1186/s42523-021-00123-2.

## Background

In beef cattle the suckling period may last 5 to 7 months, leading to a strong dam-calf bond and separation of the calf from its dam, referred to as weaning, results in both physical and psychological stress [1]. Weaning transiently increases plasma cortisol [2] and both adrenaline and noradrenaline concentrations in beef calves [2, 3], confirming calves experience a stress response following maternal separation. Stress hormones can shape the gut microbial composition, while microbial metabolites alter host physiology through modulation of neurotransmitters [4], suggesting a bi-directional interaction between host and its microbiota in the gut. It is not known, however, whether stress responses can shape or alter the composition of the upper respiratory tract (URT) microbial community. This question is of considerable importance since the bovine URT microbial community includes many opportunistic bacterial pathogens that can colonize the lung and cause pneumonia during the post-weaning period.

Although there is a limited understanding of the effects of weaning stress, epidemiological studies have implicated stress as an important factor contributing to bovine respiratory disease (BRD). Respiratory disease is the most prevalent infectious disease in weaned beef cattle, accounting for the majority of morbidity and mortality in feedlots [5]. Stressors such as transportation and co-mingling of calves from different herds are associated with an increased risk of BRD in recently weaned beef calves [6, 7]. Use of next-generation sequencing has revealed that the URT microbial community can change significantly after calves enter the feedlot [8, 9] and significant differences in the URT microbiota were also observed when comparing healthy dairy calves with age-matched cohorts diagnosed with respiratory infections [10, 11]. Studies with both beef and dairy cattle report changes in potentially pathogenic URT-resident bacterial groups (opportunistic pathogens) such as *Mannheimia (M.)*, *Pasteurella (P.)*, *Moraxella* (*Mo.*) and *Mycoplasma* (*My.*) [8–11]. These studies suggest that changes in the URT microbiome may be one factor contributing to increased lung colonization by these opportunistic pathogens. There have been no studies, however, investigating whether the stress of maternal separation (weaning) and transportation significantly perturbs the composition of the URT microbiome in a way that could contribute to increased lung colonization.

The current study focused primarily on the effect of stress on two opportunistic respiratory pathogens, *M. haemolytica* and *P. multocida*, during the 4-week post-weaning period. These two bacteria are the primary pathogens associated with fatal BRD during the first 4 weeks after suckling beef calves are weaned [12–14] and there is consistent evidence these bacteria reside in the URT of healthy calves [10]. Other opportunistic respiratory pathogens, such as *My. bovis*, are more frequently associated with chronic pneumonia or multisystemic infections later in the post-weaning period [15]. Furthermore, the effect of weaning and transportation stress on host responses to *M. haemolytica* and *P. multocida* is of substantial interest since commercial vaccines are available for these two bacteria and these vaccines are often used to immunize weaned beef calves [16]. These vaccines often fail, however, to provide significant protection against either BRD morbidity or mortality [17]. This raises the question whether stress can suppress immune responses to these bacteria or whether antibodies induced by vaccination fail to prevent respiratory infection. Therefore, we investigated whether maternal separation (weaning) and transportation significantly altered the abundance of these two bacteria in the URT and altered antibody responses to these important opportunistic respiratory pathogens.

The URT microbiome of beef calves may be altered by multiple stressors and a variety of environmental factors during the early post-weaning period. Stressors may include weaning, transportation, co-mingling of animals from multiple herds, dietary changes, a new environment, and respiratory virus infections [6]. The stress of weaning and transportation can double mortality in an experimental BRD model that combines a primary bovine herpesvirus-1 respiratory infection with a secondary *M. haemolytica* infection [18]. In this study [18], weaning and transportation significantly altered host innate immune responses during the viral and bacterial infections. Therefore, it is not known to what extent stress may contribute directly to differences reported for the URT microbiome when comparing calves with clinical signs of BRD versus healthy calves. Although a bi-directional interaction between gut microbiota and host during stress responses is well described [4, 19, 20], there is no information regarding a similar bi-direction interaction in the URT. Also, there is a lack of knowledge regarding stability of the URT microbiome when sampled repeatedly over time and the reliability of using single time point sampling to analyze URT microbial perturbations when weaned beef calves enter the feedlot.

The mucosal immune system in the URT of healthy calves responds to commensal bacteria, including opportunistic pathogens such as *M. haemolytica* and *P. multocida* [21]. It is not known, however, whether alterations in the URT microbiome or stress can influence host responses to these opportunistic pathogens. Therefore, the current study was designed to address the question whether the stress of maternal separation (weaned), by itself or combined with transportation, significantly alters either the URT microbiome or host responses to opportunistic pathogens within the microbiome. All calves in this study came from a single herd to eliminate co-mingling of animals from multiple sources. Co-mingling animals from multiple herds may contribute to URT microbiome changes and the transmission of viral respiratory pathogens. The URT microbiome was serially sampled over a one-month period to study microbiome stability in the URT of suckling beef calves and to determine whether weaning and transportation were associated with either transient or sustained changes in the URT microbiome. Expression of adrenergic receptor (ADR) genes by blood leukocytes was also monitored to determine if weaning and transportation had either short- or long-term effects on neuroimmune regulation of host responses by the stress hormones, epinephrine and norepinephrine. There are six known α-adrenergic receptors (*ADRA1A*, *ADRA1B*, *ADRA1D*, *ADRA2A*, *ADRA2B*, *ADRA2C*) and 3 known β-adrenergic receptors (*ADRB1*, *ADRB2*, *ADRB3*) but it is not known which ADRs regulate bovine immune function. Therefore, the expression of all nine ADR genes was analyzed. Serum antibody responses to the URT commensal bacteria and opportunistic pathogens, *M. haemolytica, P. multocida*, and *My. bovis* were also monitored to determine if stress altered this microbial-host interaction and whether changes in host immune response correlated with altered bacterial abundance.

## Results

### Clinical responses of calves following weaning

Elevated body temperature or fever is one of the most objective and consistent signs of undifferentiated bovine respiratory disease [22]. Therefore, rectal temperatures were measured on experimental day 0 (D0) and every third day throughout the 28 day study period. Temperatures exceeding 40 °C were considered a fever and a possible indication of respiratory infection. All calves with rectal temperatures exceeding 40 °C were examined by a clinical veterinarian for possible signs of respiratory or other infections. The physical exam included auscultation of lungs for altered respiratory sounds and a complete physical examination. All calves had temperatures below 40 °C on D0. One calf in the Weaned + Transport group had a rectal temperature of 40.2 °C and increased respiratory sounds on D3 post-weaning but the rectal temperature was below 40 °C on day 6 post-weaning. Two different calves in the Weaned + Transport group had rectal temperature of 40.2 °C and 40.3 °C on D12. One calf was diagnosed with an infection of the left hind foot (footrot) and the other calf had increased respiratory sounds. Both calves had temperature below 40 °C three days later. All other calves in the three treatment groups had rectal temperatures below 40 °C throughout the study. Monitoring rectal temperatures and physical examinations indicated no calves developed respiratory infections that required antibiotic treatment.

### Weaning, with or without transportation, alters ADR gene expression in blood leukocytes

**Fig. 1:**
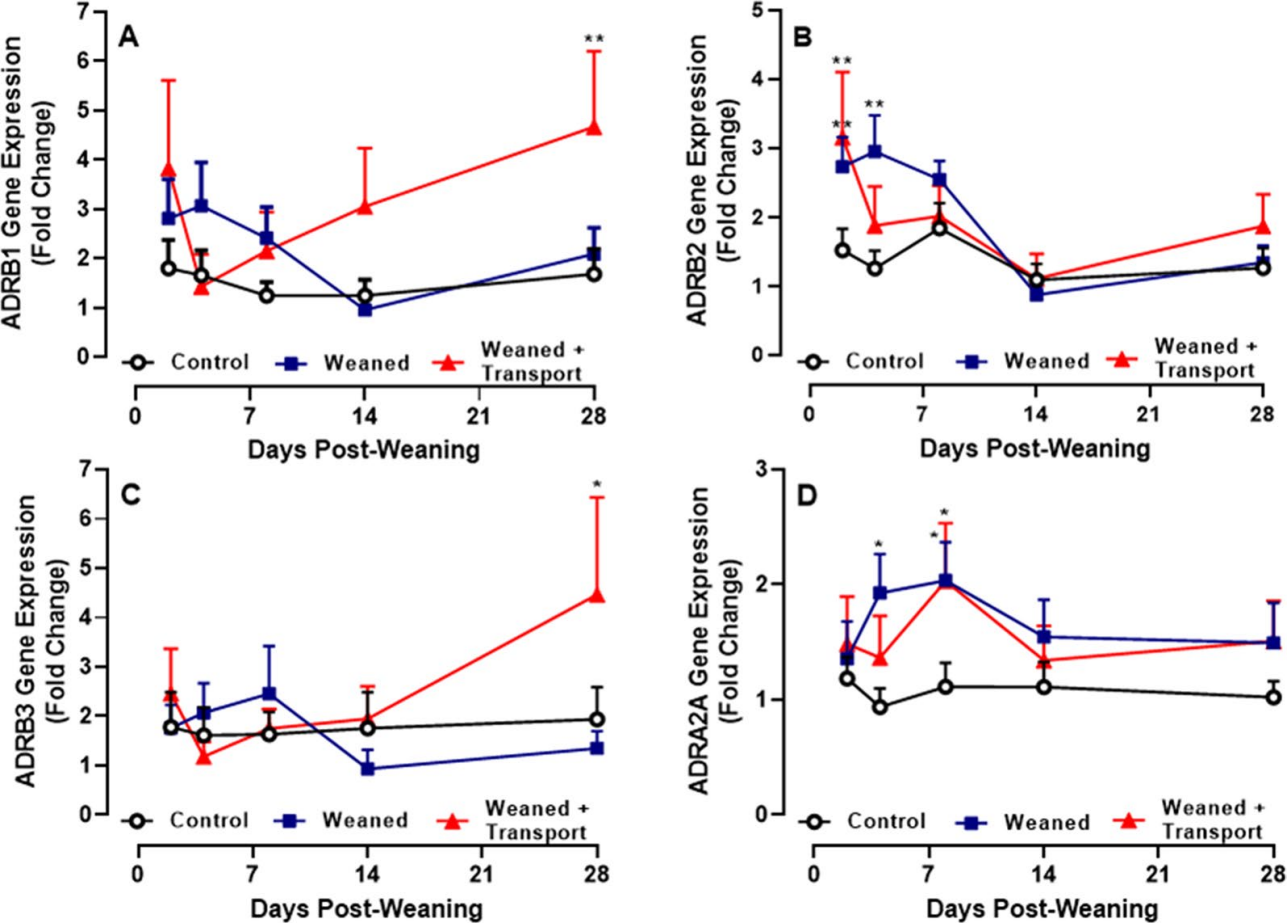
Analysis of adrenergic receptor gene expression in blood leukocytes of suckling calves (control), suckling calves separated from their dams (Weaned), and weaned and transported (Weaned + Transport) calves. Gene expression was quantified with RT-PCR and data are expressed as fold change relative to Day 0 (prior to weaning). Gene expression was analyzed for 6 alpha- and 3 beta-adrenergic receptors (ADR) and data presented are for those ADR genes where significant differences were observed when using a one-way ANOVA to compare Weaned (n = 10) and Weaned + Transport groups (n = 10) to time-matched samples from the control group (n = 10). Data presented are the mean and 1 SD *p < 0.05; **p < 0.01

Analysis of *ADR* genes expressed in bovine blood leukocytes revealed detectable levels of transcript for all 9 *ADR* genes. There were significant temporal changes in the expression of the three beta genes, *ADRB1, ADRB2,* and *ADRB3*, and one alpha gene, *ADRA2A*, when compared to time-matched samples from Suckling calves (Fig. 1). Expression of the nine *ADR* genes did not change significantly when compared over time within the group of Suckling calves. However, expression of *ADRB1* was significantly (*p* < 0.001) elevated in Weaned + Transport calves when compared to Suckling calves on D28 post-weaning (Fig. 1A). Expression of *ADRB2* was significantly (*p* < 0.05) upregulated on D2 and D4 in Weaned calves and on D2 post-weaning in Weaned + Transport calves (*p* < 0.001) when compared to time-matched samples collected from the Suckling calves (Fig. 1B). Expression of *ADRB3* tended to upregulate (*p* = 0.06) in Weaned + Transport calves when compared to the Suckling calves on D28 (Fig. 1C). Finally, expression of *ADRA2A* was significantly upregulated in Weaned calves on D4 (*p* = 0.03) and D8 (*p* = 0.05) and Weaned + Transport calves on D8 (*p* = 0.05) when compared to time-matched samples from the group of Suckling calves (Fig. 1D).

### Serum antibody responses to M. haemolytica, P. multocida, and My. bovis

**Fig. 2:**
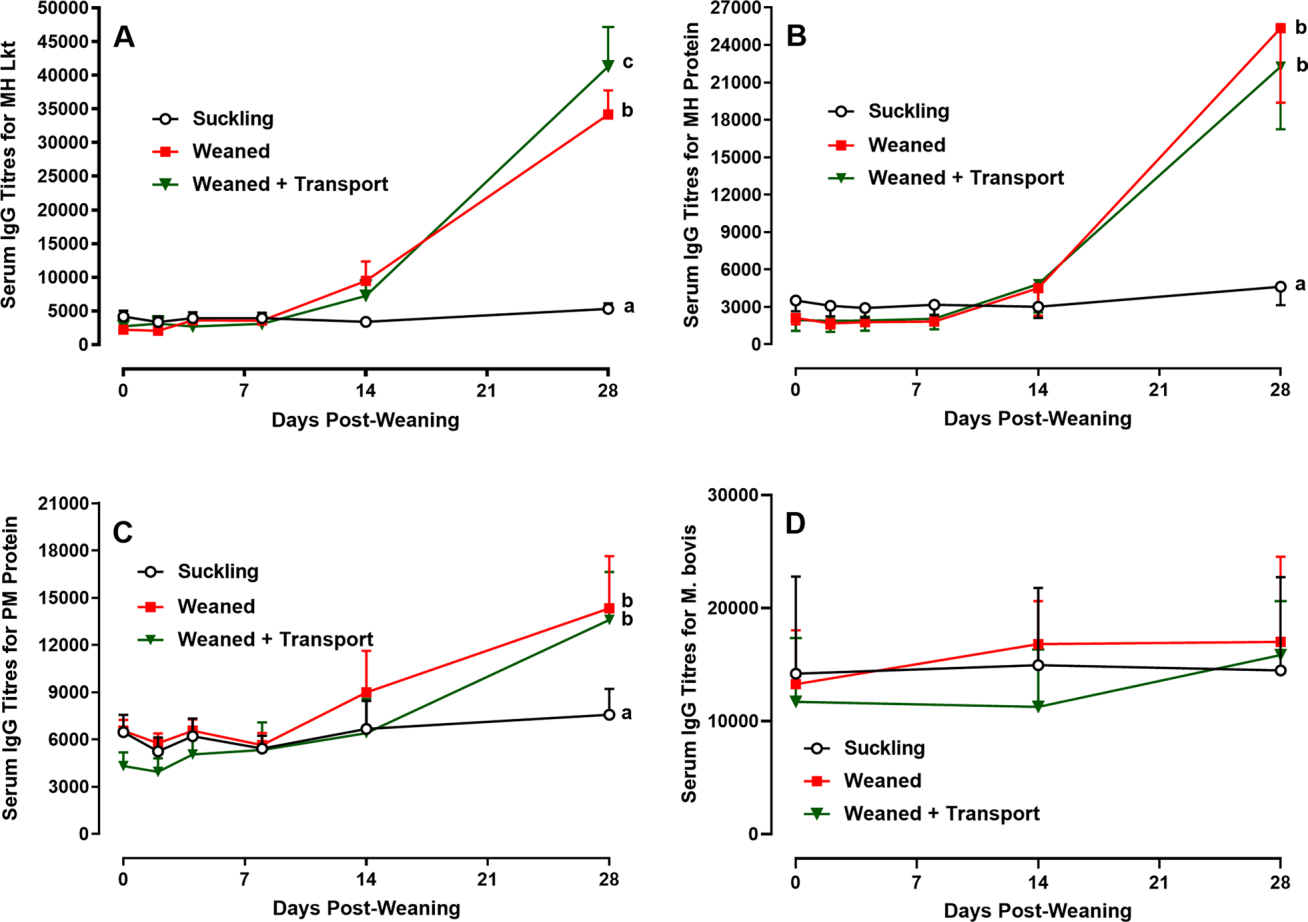
Serum antibody responses to *M. haemolytica* and *P. multocida* in suckling calves and in calves following weaning and transportation stress. Antibody titres to *M. haemolytica* were measured using both a recombinant leukotoxin (Lkt) protein (**A**) and soluble bacterial lysate (**B**). Antibody titres to *P. multocida* were measured using soluble bacterial lysate (**C**). Antibody titers to recombinant *My. bovis* MilA-ab (**D**). Significant differences were observed in antibody titres on Day 28 for *M. haemolytica* and *P. multocida*, when comparing among the three treatment groups (Suckling calves, Weaned calves, and Weaned + Transport calves) and these differences are indicated by letters (a,b,c). Data presented are the mean and 1 SD

All suckling calves had detectable but low serum antibody titers to *M. haemolytica* leukotoxin (Lkt), an important virulence factor (Fig. 2A), and bacterial lysate proteins (Fig. 2B) on experimental D0. These titers remained unchanged in the Suckling group throughout the 28-day observation period. In contrast, *M. haemolytica*-specific antibody titers were significantly (*p* < 0.01) increased on D28 (Fig. 2A, B) within both the Weaned and Weaned + Transport groups when compared to the Suckling group. The *M. haemolytica* Lkt-specific antibody titers on D28 in the Weaned + Transport group were significantly (*p* = 0.03) greater than the Weaned group.

A similar pattern in antibody responses to *P. multocida* was observed when comparing the three treatment groups (Fig. 2C). Suckling calves in all groups were seropositive for *P. multocida* on experimental D0 and antibody titers did not change significantly throughout the 28-day observation period within the Suckling group. There was, however, a significant (*p* = 0.03) increase in antibody titers specific for *P. multocida* on D28 in both the Weaned and Weaned + Transport groups when compared within each group to the D0 titers. Furthermore, the increase in antibody titers observed in these two groups on D28 were significantly (*p* = 0.02) greater when compared to the Sucking group but there was no difference when comparing Weaned and Weaned + Transport groups.

All suckling beef calves were seropositive for *My. bovis* at the beginning of the study, although there was substantial inter-animal variation in titres (Fig. 2D). There were no significant changes in *My. bovis*-specific serum antibody titres in any of the treatment groups throughout the duration of the study, Therefore, elevated serum antibody titres for *M. haemolytica* and *P. multocida* in the weaned and weaned + transport groups was not observed for *My. bovis*, another opportunistic respiratory pathogen residing in the URT.

### Colonization of the URT by opportunistic pathogens

At all time points sampled the URT of beef calves was colonized primarily by bacteria (mean relative abundance: Suckling—95.2–97.5%; Weaned—92.4–97.5%; Weaned + Transport—94.9–98.5%), followed by viruses (mean relative abundance: Suckling—0.76–3.05%; Weaned—0.64–6.73%; Weaned + Transport—0.43–3.33%) and archaea (mean relative abundance: Suckling—0.03–0.05%; Weaned—0.02–0.05%; Weaned + Transport—0.02–0.08%) (Additional file 1: Figure S1). When the microbial composition was compared at the domain level, significant temporal variations were identified from all samples regardless of the treatment group (Table 1). Comparison of the relative abundance of opportunistic pathogens at genus level revealed that the abundance of *Mannheimia*, *Pasteurella*, *Moraxella*, and *Histophilus* varied temporally regardless of treatment group (Table 1). There were no significant temporal changes in the abundance of opportunistic pathogens identified in the Suckling group. However, the median relative abundance of *Mannheimia* was highest on D4 after weaning in Weaned calves (Table 1). In Weaned + Transport calves, the median relative abundance of *Histophilus* was highest on D4, while that of Microvirus (*Enterobacteria* phage phiX174 sensu lato) was lowest on D2 (Table 1). Taxonomic assignment of assembled sequences revealed that URT microbiome of the suckling beef calves was colonized by a variety of bacteria that caused secondary infection during BRD (Additional file 2: Table S1) such as *M. haemolytica*, *P. multocida*, *H. somnii*, and *My. bovis*. The URT microbiome of these calves was also colonized by numerous other species, including *My. hyopneumoniae*, *Pseudomonas fluorescens*, *Mo. catarrhalis*, *Acinetobacter* sp. ADP1, *Actinobacillus pleuropneumoniae*, and *Psychrobacter cryohalolentis* (Additional file 2: Table S1).

**Table 1:** Differentially abundant microbial groups (metagenomics sequencing-based) in the URT of beef calves

Comparisons	Microbial group	Sampling time point (median, IQR %)						*P*-value
		D0	D2	D4	D8	D14	D28	
All samples	Bacteria (D)	97.1, 0.8^b^	97.9, 1.1^b^	97.5, 0.7^b^	96.1, 4.4^a^	97.1, 3.4^b^	97.2, 1.4^b^	0.01
	Viruses (D)	1.1, 0.9^b^	0.6, 0.6^a^	0.8, 0.6^a^	2.1, 4.7^b^	1.4, 3.2^b^	1.7, 1.4^b^	< 0.01
	Actinobacteria (P)	0.4, 0.1^a^	0.4, 0.3^a^	0.5, 0.2	0.5, 0.2	0.4, 0.2^a^	0.6,0.3^b^	< 0.01
	Bacteroidetes (P)	0.1, 0.2^a^	0.1, 0.1^a^	0.2, 0.4^a^	0.2, 0.4^a^	0.2, 0.3^a^	0.7, 1.6^b^	< 0.01
	Firmicutes (P)	0.2, 0.1^a^	0.2, 0.1^a^	0.2, 0.1^a^	0.3, 0.2	0.2, 0.2^a^	0.4, 0.4^b^	< 0.01
	Tenericutes (P)	0.5, 0.8	1.0, 1.6	0.3, 1.2	1.4, 2.2^a^	0.4, 0.7^b^	0.1, 0.2^b^	< 0.01
	*Mannheimia* (G)	0, 0.01^a^	0.02, 0.1	0.2, 0.7^b^	0.02, 0.04	0.01, 0.03^a^	0, 0.02^a^	< 0.01
	*Pasteurella* (G)	0, 0.01^a^	0.02, 0.1^b^	0.04, 0.07^b^	0.01, 0.02^b^	0, 0.02^a^	0, 0.01^a^	< 0.01
	*Moraxella* (G)	0.005, 0.02^a^	0.1, 0.2^b^	0.1, 0.3^b^	0.03, 0.1^ab^	0.01, 0.05	0.02, 0.08	0.02
	*Histophilus* (G)	0, 0.01^a^	0.02, 0.04^b^	0.06, 0.04^b^	0, 0.02^b^	0, 0.02^b^	0.005, 0.01	< 0.01
Weaned only	*Mannheimia* (G)	0, 0^a^	0.02, 0.04	0.3, 0.6^b^	0.02, 0.08	0, 0.002^a^	0.005, 0.01	< 0.01
Weaned + Transport only	*Histophilus* (G)	0.02, 0.02	0.03, 0.04	0.06, 0.02^a^	0, 0^b^	0, 0.03^b^	0.005, 0.02	0.02
	Microvirus (G)	94.8, 9.2^a^	86.7, 6.2^b^	93.9, 6.0^a^	98.5, 1.4^a^	96.4, 2.0^a^	96.9, 2.0^b^	< 0.01

D, domain (median as a % of all assigned domains); P, Phylum (median as a % of all assigned phyla within bacteria), G, Genus (median as a % of all assigned genera within bacteria or viruses); IQR, inter quartile ration

^a,b^Median with different superscripts are different at *P* < 0.05 (Post-hoc test for multiple comparisons of sampling time point using pairwise Wilcox test)

### Time-dependent variation in the URT microbial community composition

**Fig. 3:**
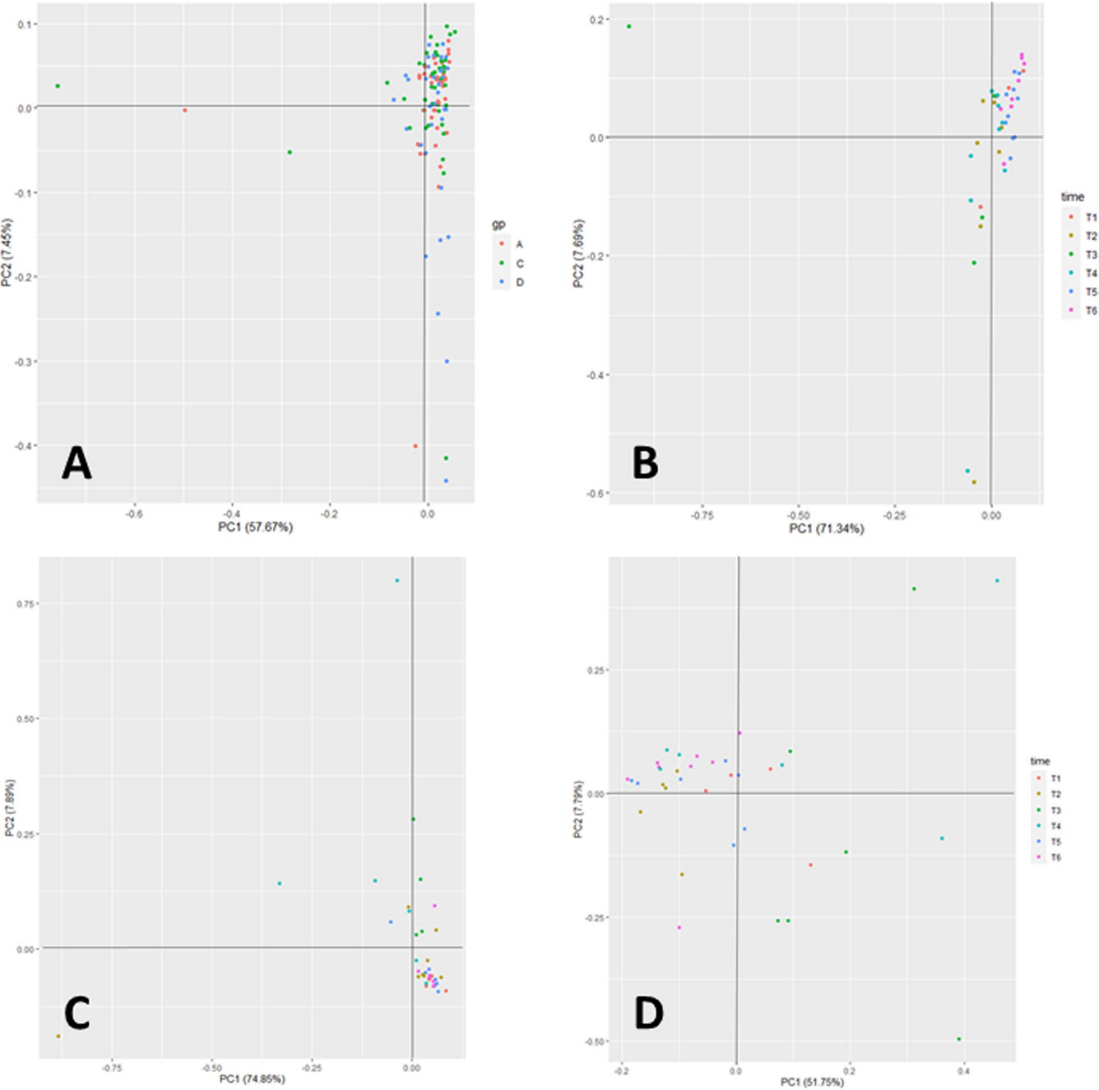
PCA-based visualization of microbial taxonomic profiles generated from the URT of beef calves. **A** All calves together comparing the effect of stressors independent of sampling point (ANOSIM R = 0.009; *p* = 0.18). **B** Suckling calves comparing sampling time (ANOSIM R = 0.2662; *p* = 1e-04). **C** Weaned calves comparing sampling time (ANOSIM R = 0.1337; *p* = 0.006). **D** Weaned + Transport calves comparing sampling time (ANOSIM R = 0.3418; *p* = 1e-04). ANOSIM-R = 0–0.25—highly overlapped; ANOSIM-R = 0.25–0.5- different with some overlap; ANOSIM-R = 0.5–0.75- different less overlap; ANOSIM-R ≥ 0.75- different. *p*-value indicates probability of ANOSIM-R

PCA plot visualization along with analysis of similarity (ANOSIM) of taxonomic profiles generated through metagenomics sequencing of samples from all calves for all time points revealed no clustering of the URT microbiome depending on the treatment group (Fig. 3A, ANOSIM-R = 0.0091; *p* = 0.18). All microbial profiles clustered closely except a few individual animals were outliers from the population at individual time points. When the same analysis approach was used within treatment groups, there was no clear separation of microbiome profiles that was dependent on sampling time point (overlapped microbiome across sampling points) in both Suckling (Fig. 3B, ANOSIM-R = 0.2662; *p* < 0.01) and Weaned calves (Fig. 3C, ANOSIM-R = 0.1337; *p* < 0.01). Time-dependent variation was more apparent in the control group than Weaned calves and fell on the border between “different with some overlap” and “highly overlapped’ categories. This variability highlights the importance of sampling a group to control for inherent temporal variability or possible effects of serial sampling on the microbiome. In contrast, the URT microbiome profiles of the Weaned + Transport calves were somewhat different (Fig. 3D, ANOSIM-R = 0.3418; *p* < 0.01) depending on the sampling point.

Comparison of the three treatment groups at individual sampling time points revealed no significant differences in the URT microbial taxonomic composition (Additional file 1: Figure S2). In addition, when the same analysis approach was used to understand the effect of stressors and sampling time on microbial functions (at KEGG orthology level 2), all functional profiles were tightly clustered for all groups at all time points (Additional file 1: Figure S3).

### Colonization of the URT by opportunistic pathogens changes with time but not with stressors

Logistic regression analysis was used to understand the relationship between colonization of the URT by opportunistic pathogens and the possible impact of stressors relative to Suckling calves. This analysis revealed the abundance of most opportunistic pathogenic bacterial genera was not linked to weaning and associated stress, except for *Haemophilus* (Table 2). Colonization by *Haemophilus* was significantly decreased in Weaned calves compared to Suckling calves, reflecting a 70% decreased likelihood of high abundance (adjusted OR 0.30, 95% CI 0.11–0.81, *p* = 0.02; Table 2) when adjusted for sampling time. In addition, Microvirus (*Enterobacteria* phage phiX174 sensu lato) colonization tended to be increased in Weaned + Transport calves compared to suckling calves, reflecting more than a two-fold increased likelihood (adjusted OR 2.32, 95% CI 0.88–6.43, *p* = 0.09; Table 2) when adjusted for sampling time.

**Table 2:** Relationship between colonization by opportunistic pathogens and bacteriophage in the URT of beef calves with weaning and transportation stressors and sampling time points

Microbial group	Factor	Comparison	Odds ratio (OR)^c^	95% CI		*P*-value
				25%	75%	
*Mannheimia*	Stressor^a^	W	0.70	0.27	1.80	0.46
		W + T	1.61	0.59	4.54	0.35
	Sampling time^b^	D2	15.04	2.91	120.85	** < 0.01**
		D4	34.85	5.10	409.30	** < 0.01**
		D8	9.79	1.96	75.76	**0.01**
		D14	9.16	1.92	68.71	**0.01**
		D28	4.99	1.03	37.16	0.07
*Pasteurella*	Stressor	W	0.06	0.36	2.47	0.90
		W + T	0.35	0.52	3.93	0.50
	Sampling time	D2	2.35	2.21	63.83	** < 0.01**
		D4	2.34	1.86	87.02	**0.01**
		D8	1.50	1.09	20.93	**0.04**
		D14	0.51	0.44	6.80	0.46
		D28	0.40	0.39	6.16	0.56
*Histophilus*	Stressor	W	1.31	0.52	3.39	0.57
		W + T	1.11	0.42	2.94	0.82
	Sampling time	D2	5.49	1.30	26.79	**0.02**
		D4	11.12	2.00	93.74	**0.01**
		D8	1.15	0.28	4.94	0.84
		D14	1.04	0.27	4.31	0.95
		D28	1.84	0.48	7.60	0.37
*Haemophilus*	Stressor	W	0.30	0.11	0.81	**0.02**
		W + T	0.67	0.25	1.80	0.43
	Sampling time	D2	9.26	1.97	56.00	** < 0.01**
		D4	50.37	6.14	1161.44	** < 0.01**
		D8	2.99	0.66	16.68	0.17
		D14	2.67	0.61	14.39	0.21
		D28	2.51	0.57	13.64	0.24
*Moraxella*	Stressor	W	1.16	0.47	2.88	0.75
		W + T	1.15	0.45	2.92	0.77
	Sampling time	D2	4.24	1.03	19.64	**0.05**
		D4	4.51	0.96	24.75	0.06
		D8	2.99	0.75	13.10	0.13
		D14	1.69	0.45	6.90	0.45
		D28	2.14	0.56	8.87	0.27
*Mycoplasma*	Stressor	W	1.91	0.79	4.76	0.15
		W + T	0.69	0.28	1.73	0.43
	Sampling time	D2	1.10	0.27	4.63	0.89
		D4	1.57	0.34	7.52	0.57
		D8	1.64	0.41	6.91	0.49
		D14	1.58	0.41	6.32	0.51
		D28	1.74	0.45	7.09	0.42
Microvirus	Stressor	W	1.57	0.62	4.10	0.35
		W + T	2.32	0.88	6.43	0.09
	Sampling time	D2	0.34	0.07	1.44	0.15
		D4	0.25	0.04	1.25	0.10
		D8	2.74	0.66	12.16	0.17
		D14	1.35	0.35	5.25	0.65
		D28	1.69	0.44	6.75	0.44
P2-like virus	Stressor	W	1.15	0.47	2.85	0.76
		W + T	0.73	0.28	1.84	0.50
	Sampling time	D2	1.85	0.46	7.78	0.39
		D4	2.69	0.57	14.16	0.22
		D8	0.51	0.12	2.04	0.34
		D14	0.94	0.25	3.54	0.92
		D28	0.73	0.19	2.80	0.65

W, Weaned calves; W + T, Weaned + Transport calves

^a^With reference to Suckling calves including all sampling time points (D0-D28)

^b^With reference to D0 sampling including all 3 treatment groups

^c^OR is calculated after adjusting logistic regression models for sampling time OR < 1 indicates a negative relationship (colonization of microbial group decreased); OR > 1 indicates a positive relationship (colonization of microbial group increased)

Colonization by bacterial genera comprised of opportunistic bacterial species, *Mannheimia*, *Pasteurella*, *Histophilus*, *Haemophilus*, and *Moraxella* displayed significant temporal variations independent of stressors (Table 2). The likelihood of colonization by *Mannheimia* increased significantly on days 2, 4, 8 and 14 when compared to D0, while likelihood of colonization by *Pasteurella* increased significantly on days 2, 4, and 8 compared to D0 (Table 2). A higher likelihood of colonization by *Histophilus* and *Haemophilus* in the URT was observed on D2 and D4 compared to D0 (Table 2). In addition, likelihood of colonization by *Moraxella* increased on D2 and tended to be increased on D4 when comparing to D0 (Table 2). The same analysis was performed using microbial colonization data after weaning (days 2, 4, 8, 14, and 28), which showed a reduction in *Haemophilus* colonization in Weaned calves compared to Suckling calves (adjusted OR 0.33, 95% CI 0.11–0.91, *p* = 0.04). A low abundance of *Haemophilus* was observed on D28 (adjusted OR 0.28, 95% CI 0.07–0.98, *p* = 0.05), whereas a lower abundance of *Histophilus* was observed on D8 and D14 than D2 (D8—adjusted OR 0.21, 95% CI 0.50–0.76, *p* = 0.02, D14—adjusted OR 0.18, 95% CI 0.05–0.66, *p* = 0.04).

When logistic regression analysis was performed within each treatment group to further understand the likelihood of colonization of opportunistic bacterial genera (stress by time interaction effect), use of neither whole microbial data (before and after treatment assignment) nor after treatment assignment (post-weaning) data displayed significant temporal variation in Suckling calves. In contrast, Weaned calves had a higher abundance of *Moraxella* on D8 (adjusted OR 25, 95% CI 1.7–1058.0, *p* = 0.04) and D28 (adjusted OR 15, 95% CI 1.4–408.4, *p* = 0.05) as well as a higher abundance of *Histophilus* on D2 (adjusted OR 14, 95% CI 1.2–384.9, *p* = 0.05) compared to D0. In Weaned + Transport calves, *Pasteurella* and *Mannheimia* tended to be higher after weaning than D0 (*Pasteurella* D8—adjusted OR 15, 95% CI 1.0–665.9, *p* = 0.08; *Mannheimia* D4—adjusted OR 15, 95% CI 0.9–665.9, *p* = 0.08; *Mannheimia* D8—adjusted OR 15, 95% CI 0.9–665.9, *p* = 0.08). Analysis of only post-weaning data revealed a lower abundance of *Mannheimia* (adjusted OR 0.11, 95% CI 0.01–0.91, p = 0.05) and *Histophilus* (adjusted OR 0.02, 95% CI 0.005–0.261, *p* = 0.01) on D14 compared to D2 only in Weaned calves but there was no temporal variation in Weaned + Transport calves.

## Microbial functions are related to weaning associated stressors

Use of logistic regression analysis to investigate the relationship between the presence/absence of a microbial function (KEGG Orthology at level 2) with weaning-associated stressors revealed that microbial functions related to “membrane transport”, “replication and repair”, and “metabolism of cofactors and vitamins” were linked to weaning (Additional file 2: Table S2). Presence of microbial functions related to “membrane transport” (adjusted OR 0.38, 95% CI 0.14–1, *p* = 0.05) and “metabolism of cofactors and vitamins” (adjusted OR 0.23, 95% CI 0.08–0.63, *p* = 0.01) displayed a decreased likelihood in Weaned calves compared to Suckling calves. In contrast, the presence of microbial functions related to “replication and repair” (adjusted OR 3.7, 95% CI 1.3–10.8, *p* = 0.01) displayed an increased likelihood in Weaned calves compared to Suckling calves. Microbial functions related to “cell motility”, “transport and catabolism”, “signal transduction”, folding, sorting and degradation”, and “transcription” varied only with time when compared to D0 (before assigning treatments) (Additional file 2: Table S2). A decreased likelihood of “membrane transport” function was evident post-weaning in both Weaned (adjusted OR 0.27, 95% CI 0.09–0.79, *p* = 0.02) and Weaned + Transport (adjusted OR 0.27, 95% CI 0.08–0.83, *p* = 0.03) calves compared to suckling calves. Microbial functions related to “replication and repair” were higher in Weaned calves (adjusted OR 3.4, 95% CI 1.1–10.6, *p* = 0.03) compared to suckling calves after weaning. “Cell communication”, “cell growth and death”, “cell mortality”, “carbohydrate metabolism”, and “xenobiotics biodegradation and metabolism” were linked only to sampling time points post-weaning (Additional file 2: Table S2).

### Weaning, with or without transportation, decreases total bacterial density

**Fig. 4:**
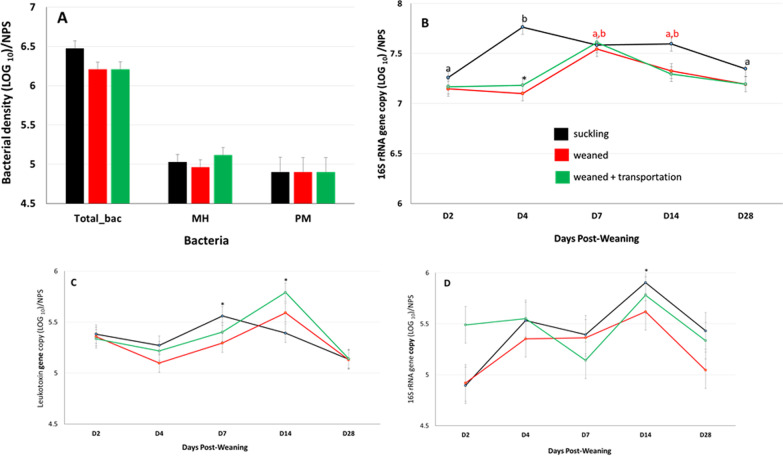
Estimated bacterial densities in the URT of beef calves. **A** Bacterial densities at the time suckling calves were assigned to treatment groups. Total bacterial density (Total_bac) and *P. multocida* (PM) density quantified as 16S rRNA gene copy/swab. *M. haemolytica* (MH) density quantified as leukotoxin (Lkt) gene copy/swab. **B** Total bacterial density after allocation of calves to the respective treatment groups, ab—represents sampling points with significantly different bacterial densities (*P* < 0.05) within suckling calves, *represents significant effect of weaning with or without transportation on bacterial density. **C** Density of *M. haemolytica* after allocation of calves to respective groups, *represents sampling points with significantly higher density of *M. haemolytica*. **D** Density of *P. multocida* after allocation of calves to the respective stressors, *represents sampling points with significantly higher density of *P. multocida*

Estimation of bacterial densities collected via deep nasopharyngeal swabs revealed that calves assigned to the three treatments groups had similar bacterial densities (D0) prior to allocating calves to treatment groups (Fig. 4A). Bacterial densities were, however, significantly lower (*p* < 0.01) in weaned calves, with (7.18 ± 0.07 log_10_ 16S rRNA gene copy/swab) or without (7.10 ± 0.07 log_10_ 16S rRNA gene copy/swab) transportation, on day 4 after weaning when compared to Suckling calves (7.76 ± 0.07 log_10_ 16S rRNA gene copy/swab) (Fig. 4B). Bacterial densities were not statistically different on D8, D14, and D28 post-weaning when comparing among treatment groups (Fig. 4B).

It is important to note that total bacterial density in URT samples also displayed temporal variation throughout the experimental period. In Suckling calves, bacterial density on D4 was higher than D2 and D28, whereas in Weaned and Weaned + Transport calves bacterial density on D8 was higher than D2, D4, and D28 (Fig. 4B).

### Density of opportunistic bacterial pathogens varies with time but not stressor

Estimation of the density of opportunistic pathogenic bacteria in the URT revealed *M. haemolytica* and *P. multocida* had colonized all suckling beef calves (Fig. 4A). Densities of *M. haemolytica* and *P. multocida* were not statistically different among the three treatment groups either before (Fig. 4A) or after allocation to treatment groups (Fig. 4C, D). Similar to total bacterial density, the density of opportunistic bacterial pathogens also displayed temporal variations during the experimental period. The highest density of *M. haemolytica* in all treatment groups was observed on D7 and D14 (Fig. 4C), while the highest density of *P. multocida* in all treatment groups was observed on D14 (Fig. 4D).

### Serum antibody responses to M. haemolytica and P. multocida are not related to bacterial abundance in the URT

A negative binomial (NB) regression analysis was used to analyze possible relationships between URT bacteria and serum antibody IgG responses to *M. haemolytica* and *P. multocida*. No significant association was identified between serum antibody responses and the relative abundance of *M. haemolytica* and *P. multocida* (Additional file 2: Table S3). Consistent with previous analyses, the NB model also revealed an increase in antibody responses with time only in Weaned and Weaned + Transport calves but not in Suckling calves (Additional file 2: Table S3).

**Fig. 5:**
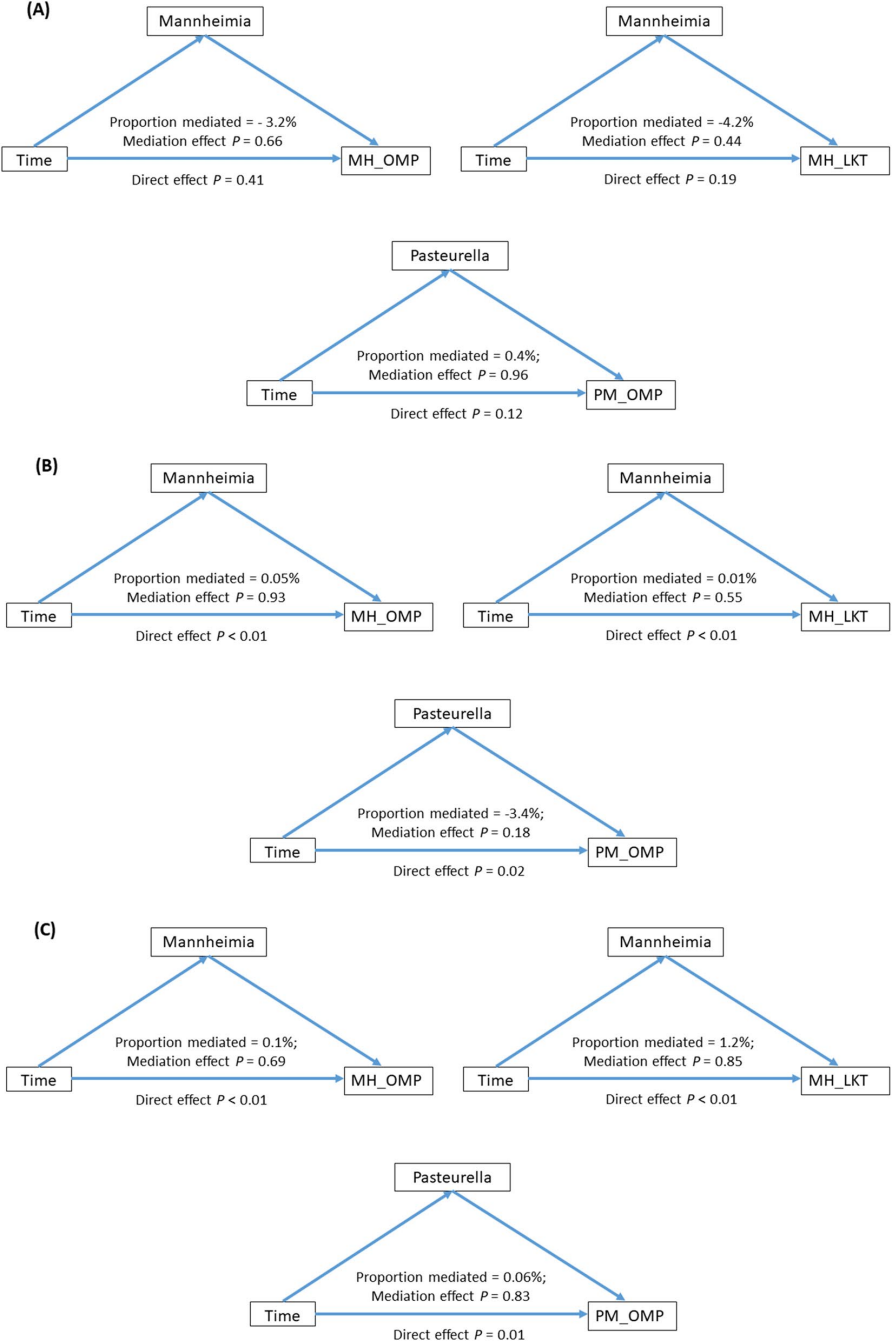
Mediation analysis to study the effect of the URT bacterial colonization on the development of antibody responses to *M. haemolytica* and *P. multocida*. **A** Mediation analysis within suckling calves. **B** Mediation analysis within Weaned calves. **C** Mediation analysis within Weaned + Transportation calves. Proportion mediated represents the proportion of the effect of the independent variable (time) on the dependent variable (antibody response) that goes through the mediator (bacterial group). Mediation effect represents the *p* value of proportion mediated. Direct effect represents the *P* value of the effect of the independent variable (time) on the dependent variable (antibody response)

Use of mediation analysis further suggested that the URT microbiota did not mediate the systemic immune responses to opportunistic pathogenic bacteria in any of the calf groups (Fig. 5). Once again mediation analysis revealed that antibody responses varied in a time dependent manner in Weaned and Weaned + Transport calves but not in Suckling calves (Fig. 5).

## Discussion

This controlled study used age-matched Suckling, Weaned, and Weaned + Transport beef calves to serially sample the URT microbiome over a one-month period. Microbiome analysis revealed the stressors of weaning and weaning + transportation had minimal effect on taxonomic and functional profiles of the URT microbiome. The microbiome of the Suckling and Weaned groups remained stable over the one-month period, while Weaned + Transport calves displayed subtle temporal changes post-weaning. These observations suggest that possibly other co-stressors, such as co-mingling, a new environment, or viral infections and the use of metaphylactic antibiotic treatment may be possible drivers of previously reported URT microbiome perturbations in beef calves during the post-weaning period. Holman and colleagues [9] analyzed the URT microbiota using samples collected prior to and after arrival in the feedlot in the absence of co-mingling and reported significant changes in the URT microbiome 2 days after arrival at the feedlot. The present study also identified significant differences in total bacterial density and the increased likelihood of high abundance of *Mannheimia*, *Pasteurella*, *Histophilus*, *Haemophilus* and *Moraxella* when comparing consecutive samples on D0 (assignment to treatment group) and D2 after weaning, regardless of the treatment. However, significant temporal effects on the likelihood of colonization of these bacteria within treatment groups were not identified, with the exception of *Moraxella* in the Weaned group. This may be a result of small sample size in the stratified logistic regression model. Therefore, changes in the likelihood of colonization with sampling time may be either stressor dependent or possibly associated with a perturbation of the URT mucosal surface during serial sampling of the microbiome. However, our logistic regression models reveal colonization by these bacterial taxa was unlikely to have been affected by the weaning and transportation stressors when compared to the suckling calves. These observations suggest a control group is important to monitor for possible effects of microbiome sampling and current data support the conclusion that the stressors of weaning, with or without transportation, had minimal effects on the URT microbiome.

The stressors of weaning and transportation in calves have been shown to increase blood cortisol [2, 18] and noradrenaline concentrations [2, 3] in beef calves. Elevated cortisol levels are associated with perturbations in both gut [21, 23] and oral microbiome [24]. Moreover, stress hormones such as epinephrine and norepinephrine, that mediate interactions between the neuroendocrine and immune system, are reported to increase gut colonization by pathogens [25]. Collectively, these studies suggest stress hormones play a role in modulating host-associated microbiomes, especially the pathobiome (opportunistic pathogens). In the present study, stress hormones in the blood of calves were not measured but expression of *ADR* genes by blood leukocytes was monitored to determine whether stress could posibly alter neuroimmune regulation of host responses. This study provides the first complete analysis of the expression of *ADR* genes in bovine blood leukocytes and significant (*p* < 0.05) stress-associated changes in the expression of *ADRB2* and *ADRA2A* were observed within the first week (day 2, 4, 8) and *ADRB1* and *ADRB3* on D28 post-weaning. These results suggest *ADRB2* and *ADRA2A* may modulate host immune responses during the first week post-weaning while *ADRB1* and *ADRB3* may modulate immune responses much later after weaning and transportation. An absence of *ADRB1* and *ADRB2* in mice promoted gut colonization by *Lactobacillaceae*, increased volatile fatty acid production, and reduced the frequency of IL17 producing T cells [26]. Microbial metabolites such as gamma-aminobutyric acid (GABA) have been shown to induce neurotransmitters [4]. Although gamma-aminobutyrate permease (a transporter of GABA) and glutamate decarboxylase (converts glutamate into GABA) were identified in the URT microbiome, their abundance did not differ among treatment groups or among sampling time points (data not shown). It may be informative to investigate further whether stress-induced changes in leukocyte *ADR* gene expression directly or indirectly alters host immune responses to opportunistic pathogens residing in the URT.

Opportunistic bacterial pathogens, such as *Mannheimia*, *Pasteurella*, and *Mycoplasma* colonize the URT microbial community of neonatal calves shortly after birth [10] indicating they are autochthonous members of the URT microbiome. Amplicon sequencing based profiling of the URT microbial community revealed a high abundance of *Mannheimia* and *Pasteurella* in neonatal [10] and older [8] calves. Metagenomics sequencing in the present study also confirmed *Mannheimia* and *Pasteurella* had colonized the URT of all suckling beef calves. However, similar to Gaeta et al. [10], who also used metagenomics sequencing, we observed the abundance of *Pseudomonas*, *Burkholderia*, *Mycoplasma*, and *Acinetobacter* in the URT microbiome exceeded that of *Mannheimia* and *Pasteurella*. *M. haemolytica* and *P. multocida* were the only species identified from the genera *Mannheimia* and *Pasteurella*, respectively through the assembled sequences. In contrast, genus *Mycoplasma* could be classified into numerous species, including *My. hyopneumoniae*, *My. conjunctivae*, *My. hyorhinis*, *My. pulmonis*, and *My. bovis*. Inclusion of no template controls in the next-generation sequencing may have further enhanced identification and classification of low frequency microbial groups by removing any sequences generated by contamination present in the molecular-grade reagents. Furthermore, comparison of the results obtained through different microbial profiling approaches suggests that microbial composition (relative abundance) can be influenced by the sequencing approach. Colonization of the URT by *Mannheimia and Pasteurella,* has attracted much attention since they are the primary cause of fatal secondary infections in the early post-weaning period [12–14]. The present study revealed all suckling calves were seropositive for *Mannheimia*, *Pasteurella* and *Mycoplasma* but in young calves, serum antibodies reacting with these opportunistic pathogens may reflect passive transfer of maternal antibody at the time of birth [20]. Maternal antibody declines as suckling calves age but the constant level of serum IgG reacting with *Mannheimia*, *Pasteurella* and *Mycoplasma* in 5- to 6-month-old suckling calves is more consistent with an active immune response to these bacteria (Fig. 2).

Following weaning, there was a significant increase in *Mannheimia-* and *Pasteurella*-specific IgG antibody titres and weaning combined with transportation resulted in an even greater increase in IgG antibody reacting with *M. haemolytica* leukotoxin (Fig. 2A). A similar increase in antibody response was not observed for *My. bovis*, another opportunistic respiratory pathogen associated primarily with pneumonia occurring much later during the post-weaning period [15]. Serum IgG antibody is generated primarily by the systemic immune system and the increase in serum antibody titres in weaned calves, with or without transportation, suggests increased systemic rather than mucosal exposure to *M. haemolytica* and *P. multocida*. These opportunistic pathogens can colonize the lungs of both healthy and diseased cattle [27], which is a possible site for systemic exposure and the induction of increased serum IgG antibody responses. The present study revealed no significant association between the density of *Mannheimia* and *Pasteurella* in the URT and serum antibody responses specific to these bacteria (Fig. 4). Therefore, the present data supports the conclusion that either increased exposure to *Mannheimia* and *Pasteurella* occurred in the lower respiratory tract of weaned and transported calves or host immune responses were altered by stress and contributed to the increased antibody responses. It should be noted, however, that altered antibody responses to *Mannheimia* and *Pasteurella* occurred despite most calves failing to develop fever or other clinical signs of respiratory disease. A previous study, using metagenomics sequencing, also revealed no significant difference in the abundance of opportunistic pathogens in the URT when comparing healthy dairy calves with calves diagnosed with BRD [11]. In contrast, following an analysis of the URT microbiome of weaned feedlot calves Timsit et al. [27] speculated that an increased abundance of opportunistic pathogens immediately after arrival at feedlots might result in increased susceptibility to BRD. Antibody responses to these bacterial pathogens were not measured in this study [27]. Thus, it is not known whether the apparent increase in bacterial abundance in this study was also associated with an increased host immune response.

## Conclusions

The present study revealed the URT microbiome is relatively stable over a one-month period in suckling beef calves and stressors, such as maternal separation (weaned) and transportation, resulted in minor microbial perturbations. When a control group was included to monitor for possible effects of microbiome sampling and inherent temporal changes in the microbiome then stress associated with maternal separation and transportation did not significantly alter the URT microbiome. Maternal separation and transportation did, however, have a marked effect on serum antibody responses to two of the opportunistic pathogens, *M. haemolytica* and *P. multocida,* that reside in the URT. No significant association was apparent between the abundance of these opportunistic pathogens in the URT and host immune responses. However, altered expression of *ADR* genes in bovine leukocytes following weaning and transportation indicate neuroimmune regulation of host responses may be altered throughout the post-weaning period. Altered immune function should be considered as a possible mechanism mediating increased host responses to bacteria residing in either the upper or lower respiratory tract. Further investigation is warranted to determine whether stress hormones can enhance host defenses against opportunistic pathogens residing in the URT.

## Materials and methods

### Animal experiment and sampling

**Fig. 6:**
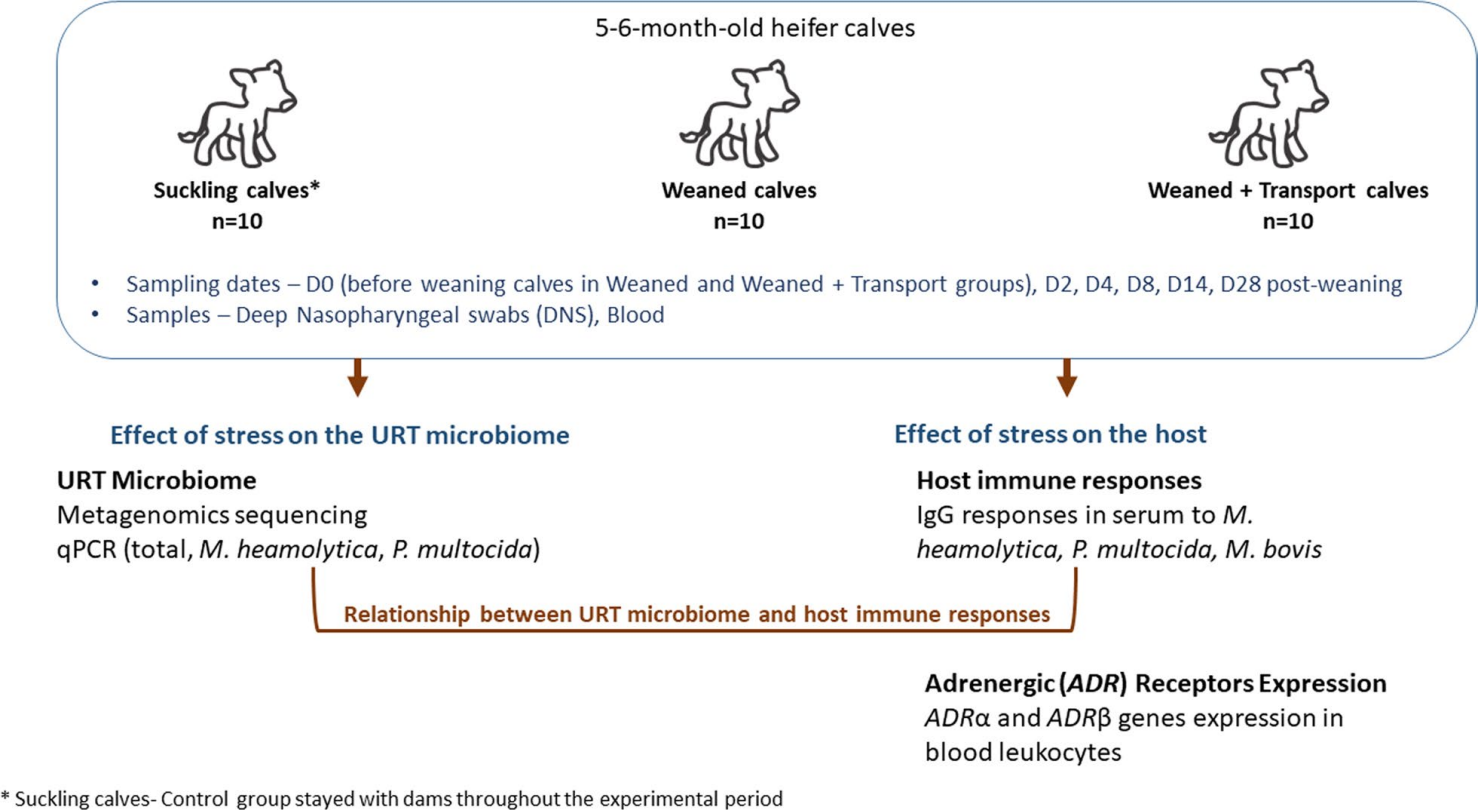
Flow chart depicting sampling process and approaches used to study the URT microbiome and host immune responses during weaning and transportation stress in beef calves

Animal experiments were completed at the University of Saskatchewan following guidelines provided by the Canadian Council on Animal Care and approved by the University of Saskatchewan Animal Care Committee (Protocol #20170015). Calves recruited to the study were 5- to 6-month-old, suckling Hereford-cross females reared within the same herd (Goodale Farm, University of Saskatchewan, Saskatoon, SK Canada). Thirty (30) calves were randomly assigned to three treatment groups using Tufts Randomization plan. Experimental groups were: Suckling calves—calves remained with dams; Weaned—calves separated from dams on experimental day 0; Weaned + Transport—calves separated from dams on experimental day 0 (D0) and transported for 4.5 h before returning to the same research facility (Fig. 6). Calves in the Weaned and Weaned + Transport group were co-mingled in a drylot with access to water and brome-alfalfa hay throughout the trial. Suckling calves remained with their dams in an adjacent paddock with access to water and brome-alfalfa hay throughout the trial. On D0, blood and deep nasal pharyngeal swabs (DNS) were collected from all calves immediately prior to separation of the Weaned and Weaned + Transport calves from their dams (Fig. 6). Blood samples for serum and leukocyte isolation was collected from the jugular vein using 10 ml BD Vacutainer™ SST and EDTA blood collection tubes (Becton Dickenson, Franklin Lakes, NJ). DNS were collected using double guarded culture swabs (Jorgensen Laboratories Inc., Loveland CO). The guarded swab was inserted into the nasal cavity a distance approximately equal to the distance from the external nares to the medial canthus of the eye. The sterile swab was then extended beyond the sheath until it contacted an obstruction and the swab was rotated three times against the mucosal surface. The swab was retracted into the protective sheath, removed from the nostril, placed in a sterile tube, transported on ice to the lab, and stored at -80 °C until DNA was extracted. Blood and DNS were collected again from all calves on days 2, 4, 8, 14, and 28 following separation of the Weaned and Weaned + Transport calves from their dams (Fig. 6). Duplicate one ml aliquots of serum and two snap-frozen pellets of 10 million blood leukocytes were stored at -20 °C and -80 °C, respectively, until analyzed.

### Isolation of blood leukocytes

Briefly, 12 mL of erythrocyte lysis buffer (0.17 M NH_4_Cl, 10 mM KHCO_3_, and 0.11 mM EDTA; pH 7.3) was added to 3 mL bovine blood. Cells were centrifuged at 325 g for 8 min and the supernatant discarded. Cell pellets were re-suspended in 1 mL Dulbecco’s Modified Eagle Medium (Sigma Aldrich) containing 10% fetal bovine serum (FBS) and cells counted with a Z2 Coulter Particle Count and Size Analyzer (Beckman Coulter, Brea, CA). Aliquots of 10 million blood leukocytes were pelleted at 311 g for 8 min and cell pellets snap-frozen in liquid nitrogen and stored at − 80 °C.

### RNA isolation from blood leukocytes

RNA was extracted from blood leukocytes using a combined TRIzol/RNEasy Mini Kit extraction method. Frozen cell pellets were suspended in 1 mL TRIzol reagent (ThermoFisher Scientific, Waltham, MA) and 200 µL chloroform (Sigma Aldrich, St. Louis, MO) was added to each sample. Samples were shaken for 15 s and incubated at room temperature for 2–3 min before centrifuging for 15 min at 13,282 g. Following centrifugation, the aqueous phase was removed, an equal amount of 70% ethanol added, and samples were added to the silica columns provided in the RNEasy Mini Kit (Qiagen, Hilden, North Rhine-Westphalia, Germany) and processed according to manufacturer’s instructions.

### cDNA synthesis and reverse transcription PCR

Synthesis of complementary DNA (cDNA) from blood leukocyte RNA template was performed following manufacturer’s instructions for the Quantitect Reverse Transcription Kit (Qiagen, Hilden, North Rhine-Westphalia, Germany). A 30-min cDNA synthesis incubation step (42 °C) was used to remove excess RNA secondary structure. Briefly, 500 ng RNA was diluted in 6 µL UltraPure DNAse/RNAse-Free Distilled Water (Invitrogen) and 1 µL of 7 × gDNA wipe-out buffer was added to remove genomic DNA. The GeneAmp 9700 PCR System (Applied Biosystems, California USA) was used to incubate this mixture for 2 min at 42 °C. Following incubation, 3 µL master mix was added to each reaction. The master mix for each reaction consisted of 2 µL 5 × Quantiscript RT buffer, 0.5 µL of primer mix, and 0.5 µL reverse transcriptase. Following addition of the master mix, each reaction was incubated for 30 min at 42 °C, followed by 3 min at 95 °C.

Reverse transcription PCR (RT-PCR) reactions were prepared with 25 ng of cDNA (5 µL of 5 ng/µL cDNA) and 10 µL of master mix. The master mix consisted of 7.5 µL 2 × PERFECTA-IQ SYBR Green Supermix (QuantaBio), 2.2 µL UltraPure DNAse/RNAse-Free Distilled Water (Invitrogen, Carlsbad, CA), and 0.3 µL of 10 µM forward and reverse primer (3 pmol; Additional file 2: Table S4). Reactions were run in Hard Shell Low-Profile 96-well semi-skirted, clear-shell, and clear-well PCR plates (BioRad, Hercules, CA). The CFX Connect Real Time System (BioRad, Hercules, CA) was used to run and quantify the real time PCR reactions. Reactions were first run at 95 °C for 2 min to activate the hot-start Taq polymerase, then for 40 cycles at 95 °C for 15 s (denature), 60 °C for 30 s (anneal), and 72 °C for 30 s (extend). Following amplification, a melt curve was applied for detection of abnormal products. The melt curve started at 65 °C, and the temperature held for 10 s before increasing by 1 °C. This pattern was repeated to a temperature of 95 °C. Results were visualized using the CFX Manager/Maestro software and corrections for primer efficiency were included in Cq value calculations.

### Serum antibody titres for M. heamolytica, P. multocida, and My. bovis

Antibody capture enzyme-linked immunosorbent assays (ELISAs) were performed to quantify serum IgG antibody titers (Fig. 6) using the protocol described in Hill et al. [22]. Multiple *Mycoplasma* spp. are present within the bovine URT but the antibody capture ELISA used was specific for *My. bovis* [28]. Briefly, proteins used for antibody capture included recombinant *M. haemolytica* leukotoxin [29], recombinant *My. bovis* MilA-ab [28] and soluble bacterial lysates prepared from *M. haemolytica* and *P. multocida*. Serum titers are presented as the inverse of the final serum dilution generating an OD reading exceeding the mean + 2 SD of the background OD value from triplicate wells containing negative serum samples.

### Profiling the URT microbiome using metagenomics sequencing

Total DNA was extracted from the DNS using PowerSoil DNA isolation kit (MO BIO Laboratory Inc., Carlsbad, CA). Briefly, the DNS was transferred into a PowerBeads tube containing the C1 solution and subjected to bead-beating using Mini-BeadBeater-16 (BioSpec Products, Bartlesville, OK) at 5000 rpm for 3 min. The supernatant was separated after centrifuging at 13,000 rpm for 15 min and subsequently used to isolate DNA following the manufacturer’s instructions. DNA quantity was measured using Qubit 3.0 fluorometer (Invitrogen, Carlsbad, CA) and Qubit dsDNA HS assay kit (ThermoFisher Scientific, Waltham, MA). Shotgun DNA libraries (Fig. 6) were prepared using NEB Ultra II DNA Library Prep Kit for Illumina (New England Biolabs Inc., Massachusetts, USA) and sequenced using Illumina HiSeq4000 PE100 (Illumina, California, USA) at Genome Quebec (McGill, Quebec).

### Analysis of metagenomics sequencing data

Demultiplexed raw data (229.8 Gb, Additional file 2: Table S5) were first run through Trimmomatic version 0.39 [30] in paired-end mode to remove adapters, low quality sequences (Phred < 20) and short sequences (< 50 bp). Then, host contamination was removed using Bowtie 2 [31], SAMtools [32] and BEDtools [33] by aligning sequences to bovine genome (UMD 3.1). Unassembled sequences with host contamination removed (53.3 Gb) were then uploaded into the MG-RAST metagenomic analysis server [34], version 4.0, and paired-ends were joined for each sample before submitting for processing. Artificial replicates, host (bovine) DNA and low-quality (Phred score < 25) sequences were removed from the raw data, and the remaining good-quality sequences were used to assign the microbial functions using the subsystems annotation source in the SEED hierarchy and KEGG Orthology and microbial taxa using RefSeq database. A maximum cut-off e-value of 1e-10, maximum identity of 70% and maximum alignment length of 80 was used as data selection criteria for the functions and taxa abundance analyses. In addition, MEGAHIT v1.1.1 [35] was used to assemble raw sequences with a minimum contig length of 200 bp and K-mer size 119. Assembled sequences were then used to assign taxonomic composition using Kraken2 database [36–38].

### Estimation of total bacteria M. haemolytica and P. multocida densities

The density of total bacteria, *M. haemolytica* and *P. multocida* were estimated using quantitative real-time PCR (qPCR) and bacterial primers (Additional file 2: Table S6). Total DNA extracted from DNS was diluted to 50 ng/µL and 1 µg template was used to perform qPCR with SYBR Green chemistry (Fast SYBR® Green Master Mix; Applied Biosystems, Foster City, CA) and StepOnePlus™ real-time PCR system (Applied Biosystems, Foster City, CA). The standard curve of total bacteria was constructed using purified PCR products amplified with 27F and 1492R primer pair, while standard curves for *M. haemolytica* and *P. multocida* were constructed using genomic DNA extracted from pure cultures of each bacterial species. Bacterial density (copy number of the 16S rRNA gene per DNS for total bacteria and *P. multocida* and copy number of leukotoxin (*lkt*) gene per DNS for *M. haemolytica*) was calculated using the following equation: (quantity mean × DNA concentration × DNA elution volume)/DNA amount used in qPCR reaction.

### Statistical analysis

The *ADR* gene expression data and antibody ELISA data were analyzed using a repeated measure model with sampling point as the repeated measure and the generalized least square function using autoregressive of order 1 (AR1) covariance structure. Significant time and treatment effects were observed and a post-hoc test for multiple comparisons of factors was performed using Tukey’s multiple comparison test to determine if there were significant treatment effects.

Taxonomic (at genus level) and functional (at KEGG Orthology level 2) compositions all metagenomes were first analyzed using principal component analysis (PCA) and Bray–Curtis dissimilarity matrix to understand the effect of stressors and sampling time point on URT microbiome. Analysis of similarities (ANOSIM) was used to test statistical significances of the PCA-based visualization. Then, the non-parametric Kruskal–Wallis test one way ANOVA by rank was performed to test the effect of stressors and sampling time point on the relative abundance of potentially pathogenic bacterial groups (*Mannheimia*, *Pasteurella*, *Haemophilus*, *Histophilus*, *Moraxella*, *Mycoplasma*) and bacteriophage (Microvirus, P2-like viruses). The same analysis was performed after stratifying data by stressor type to compare time points to test interaction effect between type of stressor and time point. A post-hoc test for multiple comparisons of factors was performed using pairwise Wilcox (Mann–Whitney U-tests) test and p values were adjusted using Benjamini and Hochberg method [31]. Data were presented as medians with 95% confidence intervals (CIs) and statistical differences were declared at p-value adjusted < 0.05.

A logistic regression analysis was performed to explore the relationship between the colonization of potential pathogens and type of stressor. High abundance of potential pathogens in the logistic regression model was defined as above (Yes) and below (NO) the median relative abundance and used to calculate the odds ratio (OR) of colonization (R package “questionr”). A stratified logistic regression model was fitted to identified temporal effect on the likelihood of colonization of microbial taxa within in each treatment group. The effect of stress on the likelihood of colonization of microbial taxa was declared in relative to suckling group after adjusting the model for sampling time. While the effect of sampling time was declared relative to D0 and D2. Associations between host immune responses to *M. haemolytica* and *P. multocida* and the abundance of genera *Mannheimia* and *Pasteurella* in the URT were explored using two different approaches. First, a negative binomial regression model was fitted for three groups together and within each stressor type. The colonization of *Mannheimia* and *Pasteurella* was defined as above (Yes) and below (NO) the median relative abundance. A mediation analysis (R package “mediation”) was then performed to test if changes in host immune responses were mediated by the respective bacterial group colonized in the URT. Bacterial densities estimated through qPCR were first normalized by log_10_ transformation and day 0 data were then analyzed using a one-way ANOVA to test effect of calf group on initial bacterial densities. Post-weaning data (days 2, 4, 8, 14 and 28) were then analyzed using a repeated measure model with sampling point as the repeated measure and the generalized least square function using autoregressive of order 1 (AR1) covariance structure, which was selected as the best fit by the Bayesian information criterion. All data were analyzed using R package (version 4.0.0).

## Supplementary Information

**Additional file 1**. Microbial profiles, taxonomic composition and functional profiles of URT microbiome.

**Additional file 2**. Bacterial species in URT and relationship between stress and microbial functions and stress and immune responses.

## Data Availability

All microbial metagenome sequence data were deposited at NCBI Sequence Read Archive (SRA) (https://www.ncbi.nlm.nih.gov/sra/PRJNA687519) under accession number PRJNA687519.

## Abbreviations

ADR: Adrenergic receptor
ANOSIM-R: Analysis of similarities R value
BRD: Bovine respiratory disease
cDNA: Complementary DNA
DNS: Deep nasopharyngeal swabs
EDTA: Ethylenediaminetetraacetic acid
ELISA: Enzyme-linked immunosorbent assay
FBS: Fetal bovine serum
IgG: Immunoglobulin G
*M.** haemolytica*: *Mannheimia haemolytica*
NB: Negative binomial
OD: Optical density
OR: Odds ratio
PCA: Principal component analysis
*P*. *multocida*: *Pasteurella multocida*
qPCR: Quantitative real-time polymerase chain reaction
RT-PCR: Reverse transcription polymerase chain reaction
rRNA: Ribosomal RNA
URT: Upper respiratory tract
