# Supplementary material for: Effect of maternal separation and transportation stress on the bovine upper respiratory tract microbiome and the immune response to resident opportunistic pathogens
Source: Anim Microbiome. 2021 Sep 19;3:62. doi: 10.1186/s42523-021-00123-2 (PMC8451078; doi:10.1186/s42523-021-00123-2)
Supplement: Supplementary file 1 — Additional file 1. Microbial profiles, taxonomic composition and functional profiles of URT microbiome. [file 42523_2021_123_MOESM1_ESM.docx]

**Supplementary Figures**

**Figure S1.** Comparison of microbial profiles generated from calves in all three groups at each sampling time point

**
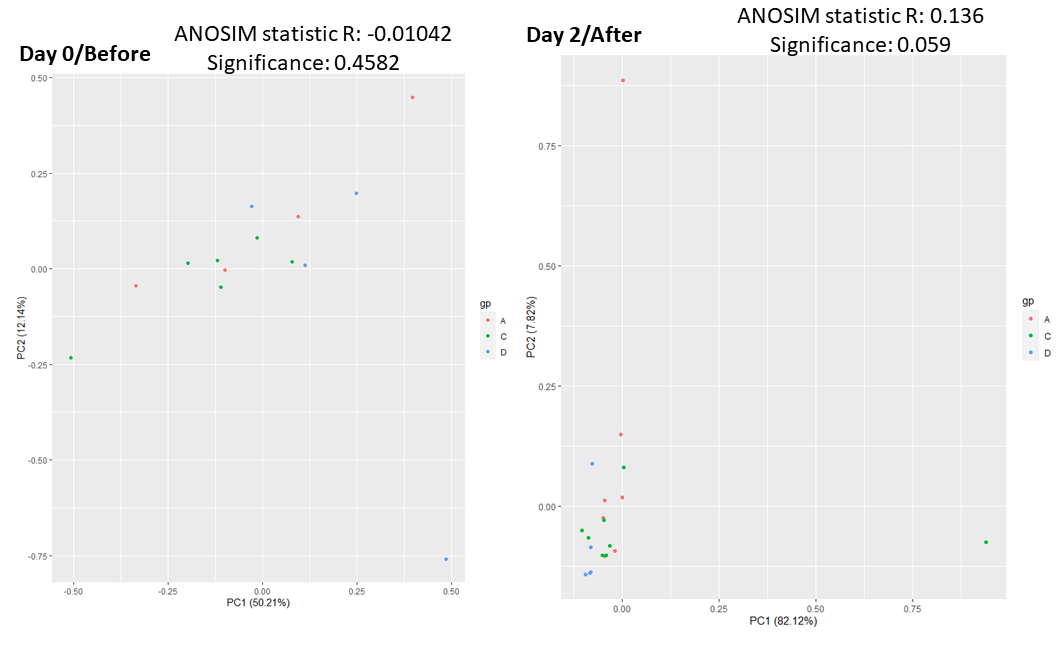
**

**
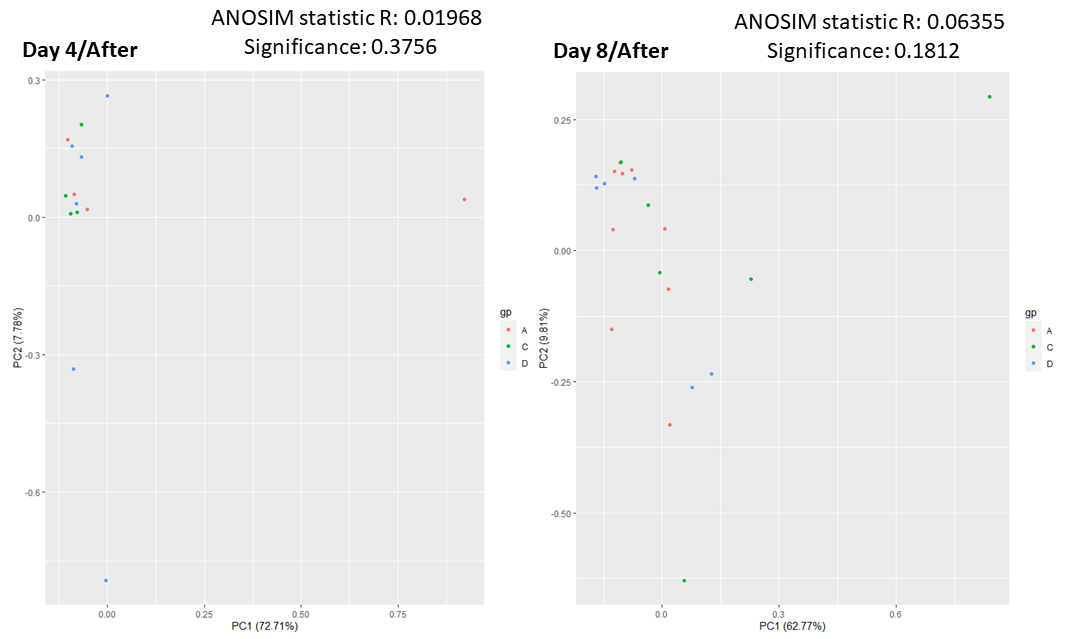
**

**
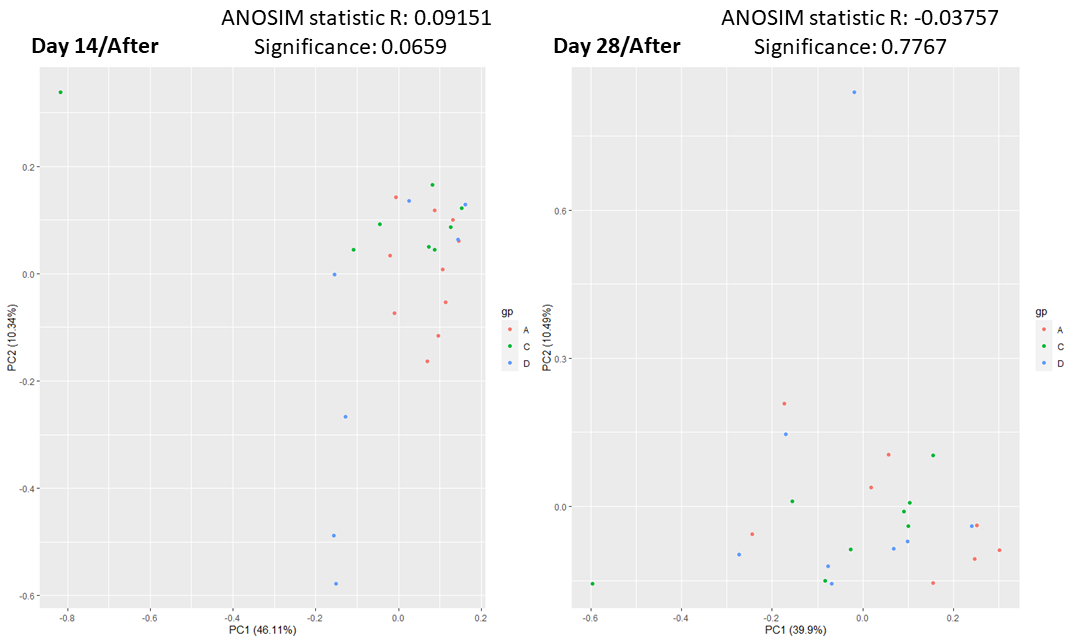
**

**Figure S2**. Taxonomic composition of the URT of beef calves. **(A)** Microbial community composition at domain level **(B)** Bacterial community composition at phylum level (**C)** Bacterial genera identified from calves at all time points (67 genera) **(D)** Viruses classified at genus level. Stack bars represents mean relative abundance of each bacterial group at each time point.

**
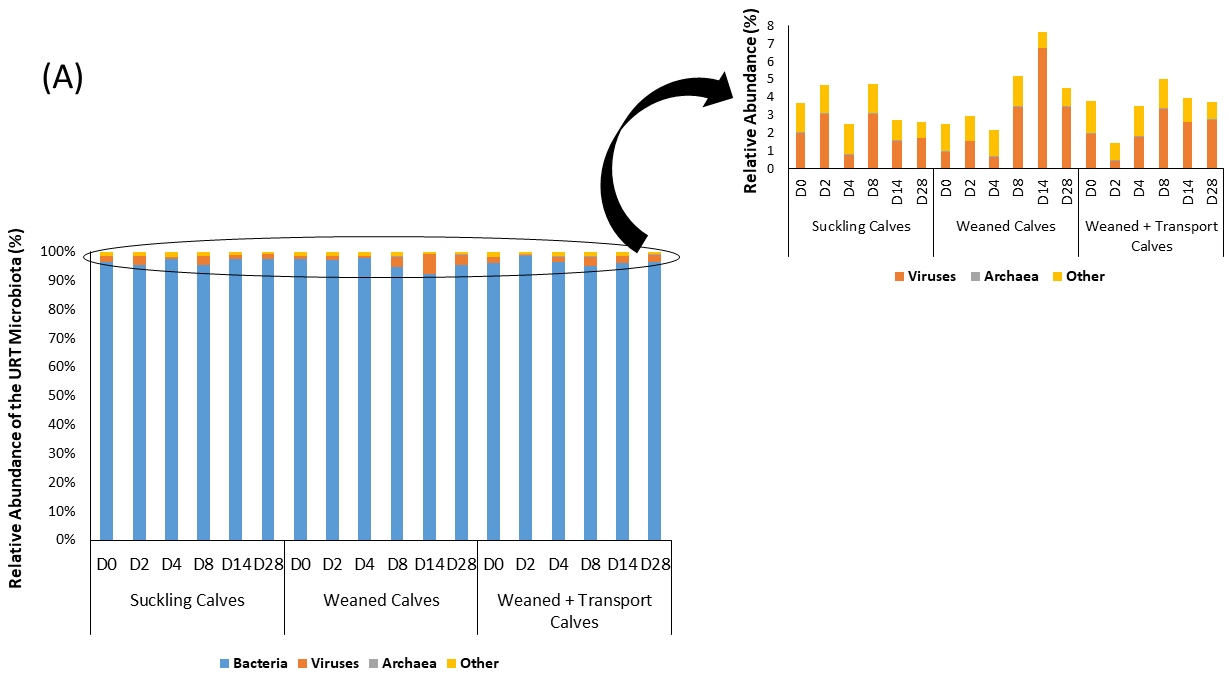
**

**
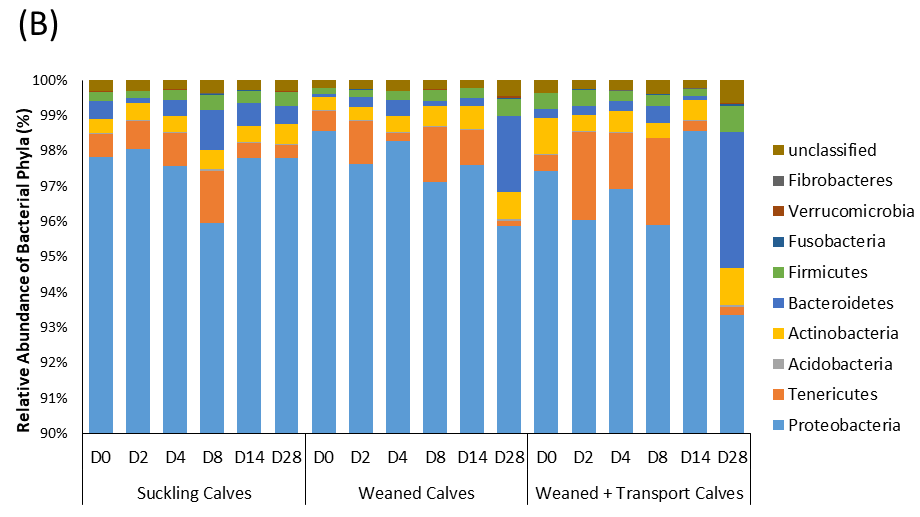
**

**
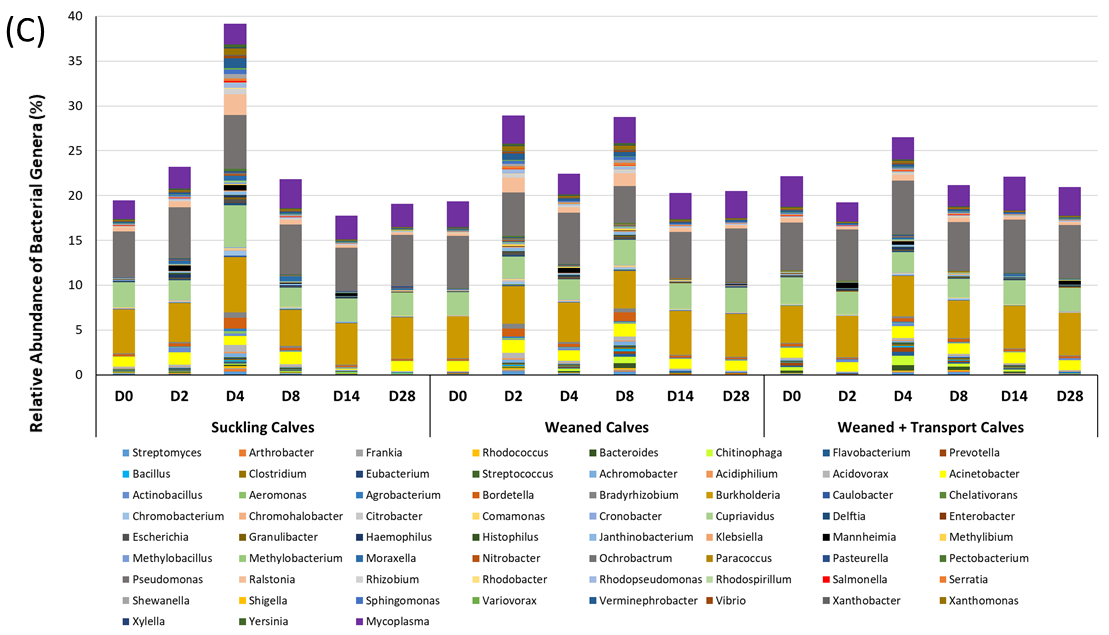
**

**
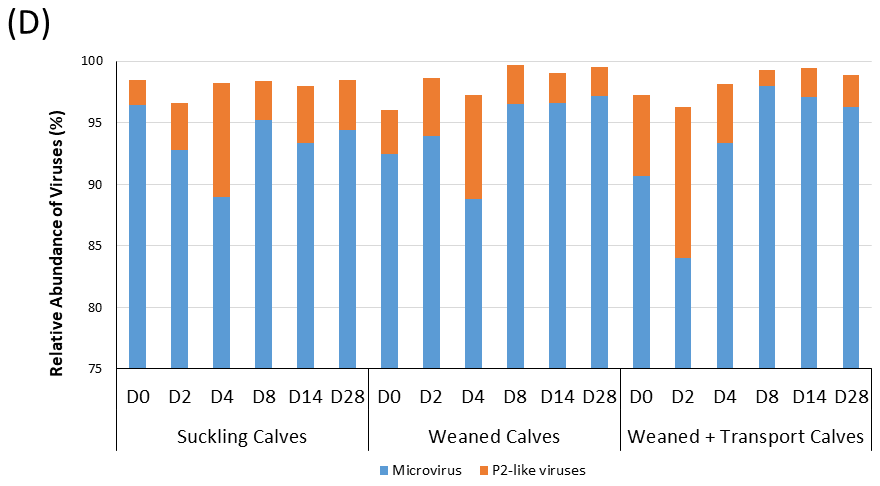
**

**Figure S4.** Microbial functional composition in the URT of beef calves (KEGG orthology level 1)

**
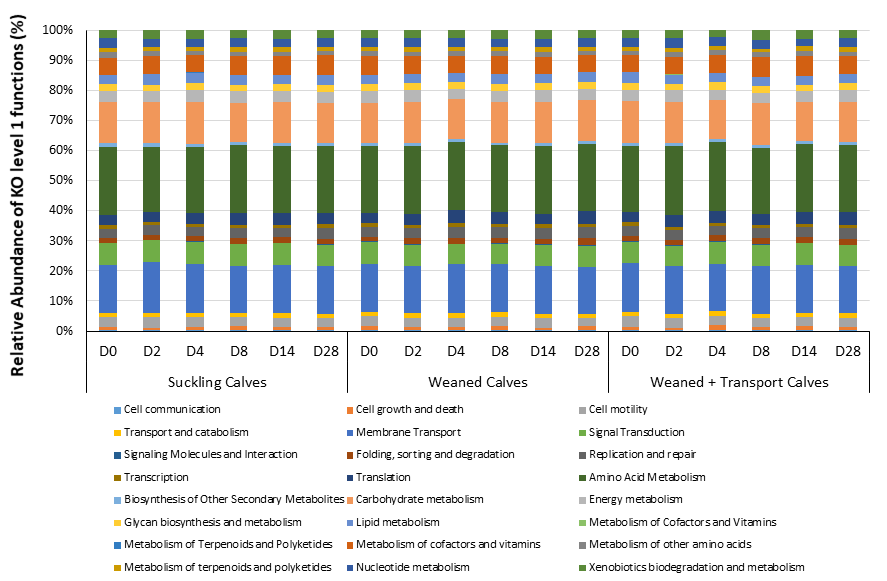
**

**Figure S3.** Comparison of the functional profiles (at KEGG orthology level 2) of the URT of beef calves


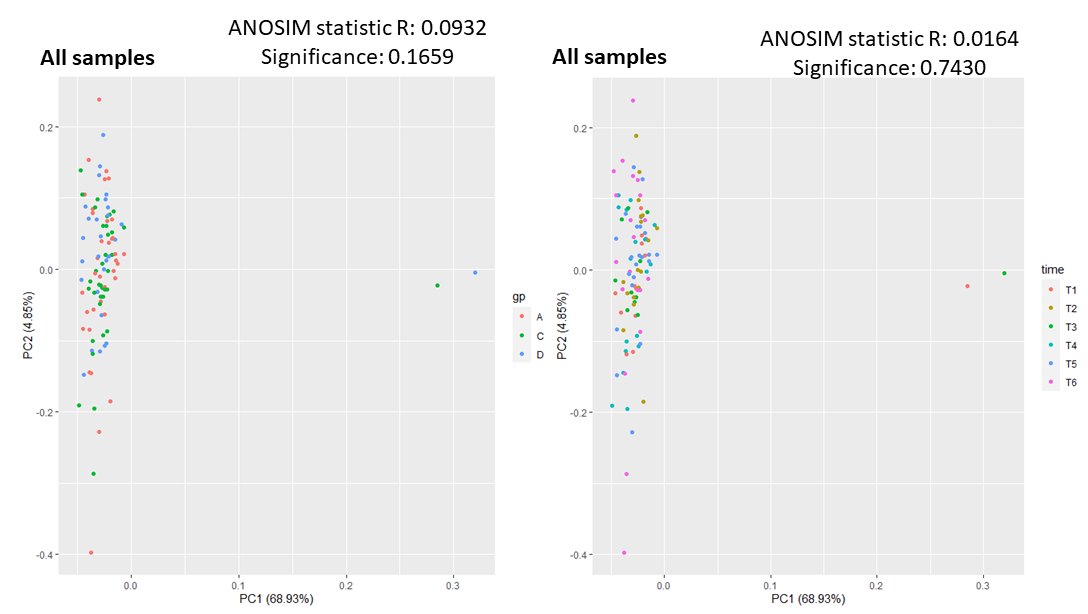


**
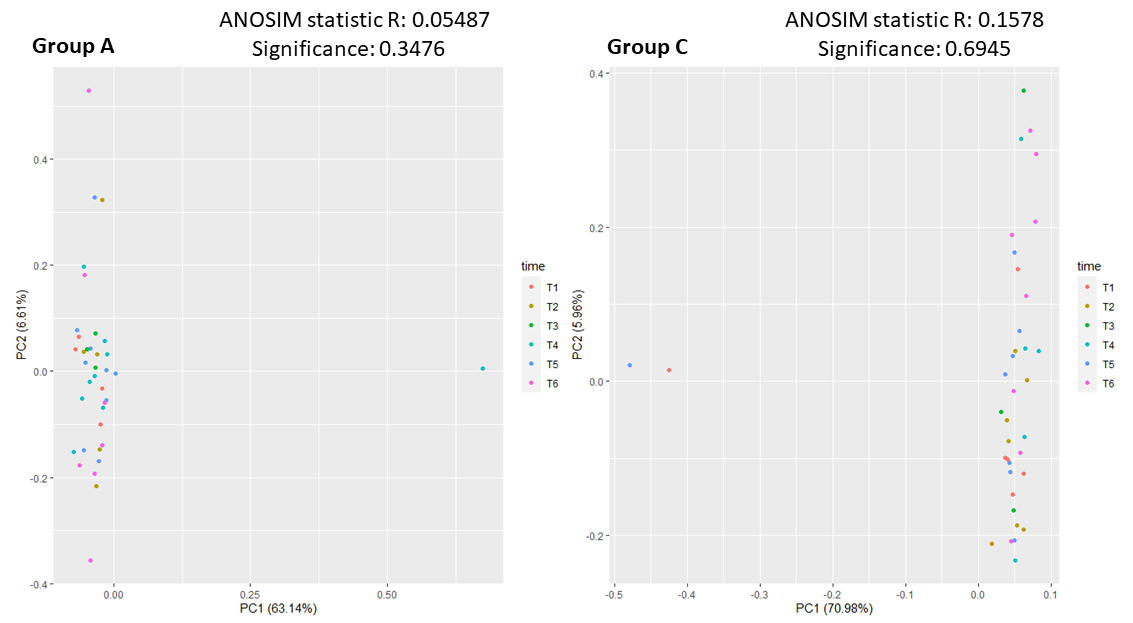
**

**
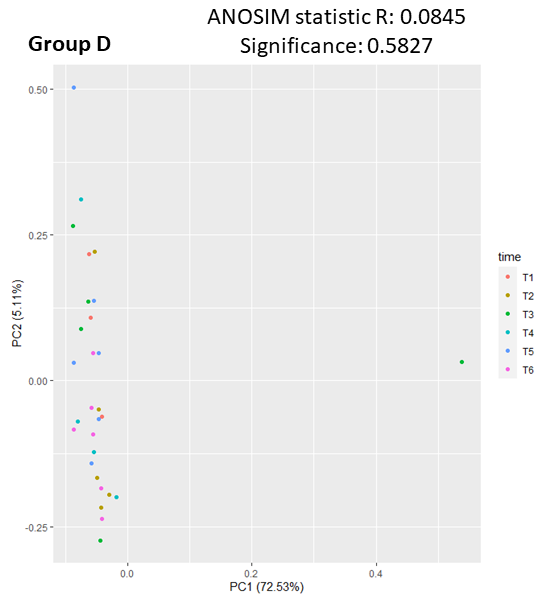
**

Supplementary Table 1. Bacterial species identified in the URT microbiome of beef calves.

| **Assembled sequences ^a^** | | **Unassembled sequences ^b^** | | | | | | | | | | | | | | | | | |
| --- | --- | --- | --- | --- | --- | --- | --- | --- | --- | --- | --- | --- | --- | --- | --- | --- | --- | --- | --- |
|  |  | **Suckling Calves** | | | | | | **Weaned Calves** | | | | | | **Weaned+Transport Calves** | | | | | |
| **Genus** | **Species** | **D0** | **D2** | **D4** | **D8** | **D14** | **D28** | **D0** | **D2** | **D4** | **D8** | **D14** | **D28** | **D0** | **D2** | **D4** | **D8** | **D14** | **D28** |
| *Acinetobacter* | *Acinetobacter sp. ADP1* | 0.093 | 0.075 | 0.055 | 0.071 | 0.103 | 0.118 | 0.061 | 0.050 | 0.068 | 0.088 | 0.060 | 0.055 | 0.117 | 0.071 | 0.064 | 0.051 | 0.069 | 0.069 |
|  | *Acinetobacter baumannii* | 0.063 | 0.045 | 0.027 | 0.060 | 0.098 | 0.157 | 0.025 | 0.044 | 0.030 | 0.034 | 0.036 | 0.029 | 0.124 | 0.060 | 0.049 | 0.027 | 0.022 | 0.048 |
| *Actinobacillus* | *Actinobacillus pleuropneumoniae* | 0.233 | 0.009 | 0.000 | 0.067 | 0.261 | 0.069 | 0.004 | 0.076 | 0.035 | 0.003 | 0.056 | 0.039 | 0.035 | 0.182 | 0.012 | 0.004 | 0.465 | 0.045 |
| *Bordetella* | *Bordetella bronchiseptica* | 0.084 | 0.092 | 0.103 | 0.108 | 0.105 | 0.092 | 0.112 | 0.086 | 0.115 | 0.077 | 0.097 | 0.094 | 0.091 | 0.116 | 0.069 | 0.110 | 0.097 | 0.104 |
| *Escherichia* | *Escherichia coli* | 0.067 | 0.076 | 0.041 | 0.064 | 0.062 | 0.062 | 0.056 | 0.053 | 0.046 | 0.041 | 0.065 | 0.069 | 0.040 | 0.049 | 0.068 | 0.044 | 0.053 | 0.068 |
| *Enhydrobacter* | *Enhydrobacter aerosaccus* | 0.104 | 0.025 | 0.009 | 0.039 | 0.408 | 0.356 | 0.012 | 0.022 | 0.038 | 0.016 | 0.027 | 0.012 | 0.237 | 0.128 | 0.095 | 0.001 | 0.013 | 0.052 |
| *Haemophilus* | *Haemophilus parasuis* | 0.098 | 0.006 | 0.000 | 0.028 | 0.094 | 0.026 | 0.001 | 0.031 | 0.006 | 0.000 | 0.030 | 0.013 | 0.014 | 0.067 | 0.004 | 0.004 | 0.130 | 0.029 |
|  | *Haemophilus influenzae* | 0.052 | 0.010 | 0.002 | 0.024 | 0.096 | 0.038 | 0.002 | 0.017 | 0.008 | 0.007 | 0.045 | 0.026 | 0.026 | 0.044 | 0.011 | 0.007 | 0.101 | 0.033 |
| *Histophilus* | *Histophilus somni* | 0.044 | 0.009 | 0.000 | 0.033 | 0.075 | 0.025 | 0.002 | 0.014 | 0.016 | 0.004 | 0.018 | 0.017 | 0.016 | 0.025 | 0.006 | 0.002 | 0.077 | 0.018 |
| *Janthinobacterium* | *Janthinobacterium sp.* | 0.091 | 0.088 | 0.103 | 0.099 | 0.095 | 0.103 | 0.095 | 0.078 | 0.095 | 0.073 | 0.091 | 0.101 | 0.090 | 0.096 | 0.101 | 0.080 | 0.095 | 0.099 |
| *Mannheimia* | *Mannheimia haemolytica* | 0.605 | 0.023 | 0.006 | 0.144 | 0.409 | 0.108 | 0.002 | 0.170 | 0.027 | 0.000 | 0.117 | 0.079 | 0.045 | 0.278 | 0.016 | 0.002 | 1.210 | 0.055 |
| *Methylibium* | *Methylibium petroleiphilum* | 0.044 | 0.043 | 0.047 | 0.049 | 0.043 | 0.042 | 0.043 | 0.050 | 0.061 | 0.044 | 0.050 | 0.049 | 0.032 | 0.041 | 0.036 | 0.046 | 0.046 | 0.047 |
| *Moraxella* | *Moraxella catarrhalis* | 0.176 | 0.149 | 0.015 | 0.125 | 0.301 | 0.477 | 0.010 | 0.056 | 0.020 | 0.057 | 0.091 | 0.032 | 0.367 | 0.094 | 0.080 | 0.002 | 0.035 | 0.078 |
| *Mycoplasma* | *Mycoplasma hyopneumoniae* | 0.391 | 0.690 | 0.820 | 1.105 | 0.300 | 0.151 | 0.489 | 1.038 | 0.424 | 0.812 | 0.744 | 0.053 | 0.340 | 1.377 | 1.413 | 1.993 | 0.806 | 0.121 |
|  | *Mycoplasma conjunctivae* | 0.037 | 0.034 | 0.048 | 0.067 | 0.019 | 0.008 | 0.028 | 0.050 | 0.027 | 0.053 | 0.050 | 0.023 | 0.046 | 0.075 | 0.063 | 0.125 | 0.043 | 0.002 |
|  | *Mycoplasma hyorhinis* | 0.003 | 0.010 | 0.006 | 0.019 | 0.008 | 0.008 | 0.010 | 0.018 | 0.002 | 0.031 | 0.009 | 0.008 | 0.013 | 0.021 | 0.022 | 0.047 | 0.017 | 0.005 |
|  | *Mycoplasma pulmonis* | 0.014 | 0.012 | 0.009 | 0.015 | 0.008 | 0.018 | 0.010 | 0.017 | 0.007 | 0.047 | 0.011 | 0.005 | 0.017 | 0.014 | 0.012 | 0.040 | 0.014 | 0.008 |
|  | *Mycoplasma alligatoris* | 0.002 | 0.006 | 0.005 | 0.011 | 0.011 | 0.027 | 0.004 | 0.007 | 0.000 | 0.088 | 0.012 | 0.005 | 0.000 | 0.010 | 0.006 | 0.031 | 0.009 | 0.011 |
|  | *Mycoplasma bovis* | 0.018 | 0.006 | 0.000 | 0.008 | 0.003 | 0.009 | 0.002 | 0.015 | 0.004 | 0.053 | 0.002 | 0.005 | 0.008 | 0.005 | 0.002 | 0.014 | 0.004 | 0.003 |
| *Ochrobactrum* | *Ochrobactrum anthropi* | 0.043 | 0.037 | 0.033 | 0.030 | 0.034 | 0.042 | 0.044 | 0.042 | 0.026 | 0.038 | 0.033 | 0.042 | 0.056 | 0.042 | 0.030 | 0.045 | 0.042 | 0.034 |
| *Pasteurella* | *Pasteurella multocida* | 0.019 | 0.017 | 0.002 | 0.051 | 0.198 | 0.031 | 0.005 | 0.014 | 0.002 | 0.010 | 0.289 | 0.011 | 0.084 | 0.016 | 0.008 | 0.005 | 0.066 | 0.022 |
| *Polaromonas* | *Polaromonas sp. JS666* | 0.140 | 0.141 | 0.120 | 0.132 | 0.121 | 0.117 | 0.128 | 0.124 | 0.136 | 0.129 | 0.139 | 0.109 | 0.132 | 0.132 | 0.111 | 0.153 | 0.141 | 0.125 |
|  | *Pseudomonas fluorescens* | 0.352 | 0.325 | 0.379 | 0.358 | 0.321 | 0.380 | 0.340 | 0.353 | 0.370 | 0.323 | 0.329 | 0.358 | 0.317 | 0.333 | 0.341 | 0.330 | 0.373 | 0.354 |
| *Pseudomonas* | *Pseudomonas aeruginosa* | 0.317 | 0.291 | 0.256 | 0.285 | 0.267 | 0.278 | 0.272 | 0.250 | 0.295 | 0.264 | 0.251 | 0.257 | 0.326 | 0.279 | 0.217 | 0.320 | 0.276 | 0.261 |
| *Psychrobacter* | *Psychrobacter cryohalolentis* | 0.069 | 0.051 | 0.004 | 0.040 | 0.171 | 0.253 | 0.010 | 0.026 | 0.020 | 0.023 | 0.033 | 0.014 | 0.169 | 0.067 | 0.046 | 0.003 | 0.008 | 0.029 |
|  | *Psychrobacter arcticus* | 0.081 | 0.044 | 0.007 | 0.037 | 0.146 | 0.188 | 0.012 | 0.015 | 0.015 | 0.022 | 0.028 | 0.012 | 0.107 | 0.057 | 0.029 | 0.006 | 0.013 | 0.035 |
|  | *Psychrobacter sp. PRwf-1* | 0.136 | 0.072 | 0.011 | 0.057 | 0.246 | 0.344 | 0.016 | 0.026 | 0.025 | 0.035 | 0.055 | 0.023 | 0.220 | 0.083 | 0.071 | 0.009 | 0.018 | 0.064 |
| *Ralstonia* | *Ralstonia solanacearum* | 0.316 | 0.313 | 0.340 | 0.332 | 0.342 | 0.327 | 0.311 | 0.315 | 0.318 | 0.340 | 0.333 | 0.303 | 0.290 | 0.315 | 0.323 | 0.377 | 0.337 | 0.305 |
|  | *Ralstonia pickettii* | 0.050 | 0.072 | 0.078 | 0.073 | 0.083 | 0.082 | 0.073 | 0.078 | 0.078 | 0.059 | 0.068 | 0.074 | 0.082 | 0.071 | 0.062 | 0.072 | 0.085 | 0.063 |
| *Rhodopirellula* | *Rhodopirellula baltica* | 0.006 | 0.006 | 0.000 | 0.005 | 0.007 | 0.002 | 0.002 | 0.001 | 0.004 | 0.014 | 0.003 | 0.009 | 0.000 | 0.005 | 0.005 | 0.004 | 0.005 | 0.006 |

^a^ Bacterial species identified using Kraken 2 database

^b^ mean relative abundance (%) of identified species using RefSeq database in MG-RAST

Supplementary Table 2. Relationship between the presence of a microbial function (KEGG Orthology at level 2) in the URT of beef calves with weaning and transportation stressors and sampling time points.

| **Microbial function** | **All samples** | | | | | **After Weaning** | | | | |
| --- | --- | --- | --- | --- | --- | --- | --- | --- | --- | --- |
|  | **Factor^a^** | **OR** | **25%** | **97.5%** | **P-value** | **Factor^b^** | **OR** | **25%** | **97.5%** | **P-value** |
| Cell communication |  |  |  |  |  | D4 | 4.29 | 0.84 | 26.72 | 0.09 |
|  |  |  |  |  |  | D8 | 4.09 | 1.00 | 18.94 | 0.06 |
| Cell growth and death |  |  |  |  |  | **D4** | **12.68** | **2.16** | **112.38** | **0.01** |
|  |  |  |  |  |  | D8 | 3.60 | 0.86 | 17.38 | 0.09 |
|  |  |  |  |  |  | **D28** | **5.29** | **1.35** | **24.50** | **0.02** |
| Cell motility | **D28** | **0.22** | **0.04** | **0.91** | **0.04** | D28 | 0.27 | 0.07 | 1.01 | 0.06 |
| Transport and catabolism | D4 | 5.15 | 0.91 | 36.4 | 0.08 |  |  |  |  |  |
|  | D28 | 3.71 | 0.88 | 18.0 | 0.08 |  |  |  |  |  |
| Membrane Transport | **W** | **0.38** | **0.14** | **1.00** | **0.05** | **W** | **0.27** | **0.09** | **0.79** | **0.02** |
|  | W+T | 0.40 | 0.14 | 1.10 | 0.08 | **W+T** | **0.27** | **0.08** | **0.83** | **0.03** |
| Signal Transduction | D2 | 0.23 | 0.04 | 1.04 | 0.07 |  |  |  |  |  |
|  | D28 | 0.26 | 0.05 | 1.11 | 0.08 |  |  |  |  |  |
| Folding, sorting and degradation | **D2** | **22.3** | **3.21** | **460** | **0.01** |  |  |  |  |  |
|  | **D4** | **28.8** | **3.38** | **670** | **0.01** |  |  |  |  |  |
|  | **D8** | **10.7** | **1.55** | **219** | **0.04** |  |  |  |  |  |
|  | D14 | 8.38 | 1.28 | 167 | 0.06 |  |  |  |  |  |
|  | **D28** | **24.3** | **3.67** | **491** | **0.01** |  |  |  |  |  |
| Replication and repair | **W** | **3.70** | **1.35** | **10.8** | **0.01** | **W** | **3.35** | **1.14** | **10.55** | **0.03** |
|  |  |  |  |  |  | D14 | 3.24 | 0.85 | 13.50 | 0.09 |
|  |  |  |  |  |  | **D28** | **5.30** | **1.33** | **23.87** | **0.02** |
| Transcription | **D8** | **6.33** | **1.32** | **38.4** | **0.03** |  |  |  |  |  |
|  | D14 | 4.01 | 0.91 | 22.1 | 0.08 |  |  |  |  |  |
|  | **D28** | **5.40** | **1.21** | **30.5** | **0.04** |  |  |  |  |  |
| Carbohydrate metabolism |  |  |  |  |  | **D8** | **0.23** | **0.05** | **0.92** | **0.04** |
| Metabolism of cofactors and vitamins | **W** | **0.23** | **0.08** | **0.63** | **0.01** | **W** | **0.12** | **0.03** | **0.37** | **<0.01** |
|  | **D14** | **10.6** | **1.98** | **74.4** | **0.01** | D8 | 4.85 | 0.99 | 27.74 | 0.06 |
|  | D28 | 4.58 | 0.99 | 26.5 | 0.06 |  |  |  |  |  |
| Metabolism of other amino acids |  |  |  |  |  | W+T | 2.74 | 0.90 | 9.08 | 0.08 |
| Xenobiotics biodegradation and metabolism |  |  |  |  |  | D8 | 3.54 | 0.88 | 15.75 | 0.08 |
|  |  |  |  |  |  | **D14** | **4.06** | **1.09** | **16.75** | **0.04** |

^a^ Treatment effect is calculated with reference to Suckling calves including all sampling time points (D0-D28) and Time effect is calculated with reference to D0 sampling including all 3 treatment groups

^b^ Treatment effect is calculated with reference to Suckling calves including all sampling time points after weaning (D2-D28) and Time effect is calculated with reference to D2 sampling including all 3 treatment groups

OR < 1 indicates a negative relationship (colonization of microbial group decreased); OR > 1 indicates a positive relationship (colonization of microbial group increased)

W –Weaned calves; W+T – Weaned+Transport calves

Supplementary Table 3. Relationship between antibody responses and bacterial colonization

| **Comparison** | **Antibodies** | **Variable** | **Estimate** | **Std. Error** | ***P* - value** |
| --- | --- | --- | --- | --- | --- |
| All | MH_OMP | *Mannheimia* | 0.19 | 0.15 | 0.20 |
|  |  | W | -0.20 | 0.20 | 0.33 |
|  |  | W+T | -0.07 | 0.21 | 0.75 |
|  |  | D2 | -0.08 | 0.32 | 0.80 |
|  |  | D4 | 0.33 | 0.35 | 0.34 |
|  |  | D8 | 0.24 | 0.31 | 0.45 |
|  |  | **D14** | **0.66** | **0.30** | **0.03** |
|  |  | **D28** | **2.13** | **0.31** | **< 0.01** |
|  | MH_LKT | *Mannheimia* | 0.16 | 0.15 | 0.27 |
|  |  | W | 0.21 | 0.20 | 0.29 |
|  |  | W+T | -0.03 | 0.20 | 0.90 |
|  |  | D2 | 0.41 | 0.31 | 0.19 |
|  |  | D4 | 0.40 | 0.34 | 0.24 |
|  |  | D8 | 0.37 | 0.31 | 0.23 |
|  |  | **D14** | **0.96** | **0.30** | **< 0.01** |
|  |  | **D28** | **2.26** | **0.30** | **< 0.01** |
|  |  | *Pasteurella* | 0.60 | 0.47 | 0.20 |
|  | PM_OMP | W | 0.09 | 0.15 | 0.54 |
|  |  | W+T | -0.28 | 0.16 | 0.07 |
|  |  | D2 | -0.10 | 0.24 | 0.69 |
|  |  | D4 | 0.11 | 0.26 | 0.68 |
|  |  | D8 | 0.15 | 0.24 | 0.52 |
|  |  | D14 | 0.31 | 0.23 | 0.17 |
|  |  | **D28** | **0.89** | **0.23** | **< 0.01** |
| Suckling Calves | MH_OMP | *Mannheimia* | 0.28 | 0.18 | 0.12 |
|  |  | D2 | -0.49 | 0.49 | 0.33 |
|  |  | D4 | -0.08 | 0.55 | 0.89 |
|  |  | D8 | -0.20 | 0.46 | 0.66 |
|  |  | D14 | -0.14 | 0.46 | 0.77 |
|  |  | D28 | 0.22 | 0.47 | 0.64 |
|  | MH_LKT | *Mannheimia* | 0.23 | 0.16 | 0.15 |
|  |  | D2 | -0.12 | 0.44 | 0.79 |
|  |  | D4 | 0.04 | 0.49 | 0.93 |
|  |  | D8 | -0.12 | 0.41 | 0.77 |
|  |  | D14 | 0.10 | 0.41 | 0.81 |
|  |  | D28 | 0.20 | 0.42 | 0.64 |
|  | PM_OMP | *Pasteurella* | 0.63 | 0.57 | 0.27 |
|  |  | D2 | 0.04 | 0.44 | 0.93 |
|  |  | D4 | 0.34 | 0.50 | 0.50 |
|  |  | D8 | 0.40 | 0.42 | 0.34 |
|  |  | D14 | 0.59 | 0.41 | 0.15 |
|  |  | D28 | 0.57 | 0.43 | 0.19 |
| Weaned Calves | MH_OMP | *Mannheimia* | -0.04 | 0.46 | 0.92 |
|  |  | D2 | 0.22 | 0.37 | 0.56 |
|  |  | D4 | -0.13 | 0.51 | 0.79 |
|  |  | D8 | 0.26 | 0.39 | 0.51 |
|  |  | D14 | 0.47 | 0.36 | 0.20 |
|  |  | **D28** | **2.75** | **0.37** | **< 0.01** |
|  | MH_LKT | *Mannheimia* | -0.63 | 0.51 | 0.21 |
|  |  | D2 | 0.91 | 0.40 | 0.02 |
|  |  | D4 | 0.72 | 0.56 | 0.20 |
|  |  | D8 | 0.59 | 0.43 | 0.17 |
|  |  | **D14** | **1.28** | **0.40** | **< 0.01** |
|  |  | **D28** | **2.76** | **0.40** | **< 0.01** |
|  | PM_OMP | *Pasteurella* | 0.73 | 0.85 | 0.39 |
|  |  | D2 | -0.03 | 0.30 | 0.93 |
|  |  | D4 | 0.25 | 0.34 | 0.46 |
|  |  | D8 | 0.04 | 0.30 | 0.90 |
|  |  | D14 | 0.22 | 0.28 | 0.43 |
|  |  | **D28** | **0.98** | **0.28** | **< 0.01** |
| Weaned + Transport Calves | MH_OMP | *Mannheimia* | 0.09 | 0.26 | 0.74 |
|  |  | D2 | -0.15 | 0.64 | 0.81 |
|  |  | D4 | 1.42 | 0.61 | 0.02 |
|  |  | D8 | 1.00 | 0.61 | 0.10 |
|  |  | **D14** | **2.01** | **0.59** | **< 0.01** |
|  |  | **D28** | **3.21** | **0.58** | **< 0.01** |
|  | MH_LKT | *Mannheimia* | 0.37 | 0.25 | 0.14 |
|  |  | D2 | 0.06 | 0.64 | 0.92 |
|  |  | D4 | 1.08 | 0.61 | 0.07 |
|  |  | D8 | 0.97 | 0.60 | 0.11 |
|  |  | **D14** | **1.92** | **0.59** | **< 0.01** |
|  |  | **D28** | **3.45** | **0.58** | **< 0.01** |
|  | PM_OMP | *Pasteurella* | 0.01 | 1.68 | 1.00 |
|  |  | D2 | -0.34 | 0.51 | 0.51 |
|  |  | D4 | -0.18 | 0.51 | 0.72 |
|  |  | D8 | -0.05 | 0.49 | 0.92 |
|  |  | D14 | 0.09 | 0.49 | 0.86 |
|  |  | **D28** | **1.00** | **0.47** | **0.03** |

^a^ With reference to Suckling calves

^b^ With reference to D0 sampling

^c^ A negative estimate represents one unit increase in bacterial relative abundance decreased log count of antibody responses or stressors/postweaning time point has lower antibody responses than reference. A positive represents one unit increase in bacterial relative abundance increased log count of antibody responses or stressors/postweaning time point has higher antibody responses than reference.

Supplementary Table 4. Validated Primers for Bovine *ADR* Genes

| **Gene (Accession number)** | **Primer Sequence** | **Product Size**  **(bp)** | **Primer Efficiency (%)** |
| --- | --- | --- | --- |
| *ADRB1* | F 5’ – CCTAGCCAACGTGGTGAAGG - 3’  R 5’ – CTGCGGCAGTAGATGATGGG -3’ | 117 | 90.8 |
| *ADRB2* | F 5’ – TAGCCATCACGTCACCCTTC - 3’  R 5’ – AAGGAGGTAAGGCCAGACAC -3’ | 100 | 93.6 |
| *ADRB3* | F 5’ – TTCTGCCTCTGCGGGAACAC - 3’  R 5’ – GCACCACGTTGACCACAAAG -3’ | 99 | 91.3 |
| *ADRA1A* | F 5’ – GATTTCAGGCCCTCAGAAAC – 3’  R 5’ – GCAGACACTGGATTCTCAAG – 3’ | 142 | 88.5 |
| *ADRA1B* | F 5’ – CCCTTCTATGCCCTCTTCTC – 3’  R 5’ – GGTTCTTGGTGGTCCTCTTG – 3’ | 109 | 91.7 |
| *ADRA1D* | F 5’ – CTTCCTGGCAGCCTTCATCC – 3’  R 5’ – GGAGCACTGTGGCACTTAGC – 3’ | 144 | 89.6 |
| *ADRA2A* | F 5’ – CATCGGAGTGTTCGTGGTGT – 3’  R 5’ – AGCTGTTGCAGTAGCCGAAC – 3’ | 122 | 90.9 |
| *ADRA2B* | F 5’ – GGTCTACCTGCGCATCTACC – 3’  R 5’ – GGTGTGGCTGCTTAGACTCC – 3’ | 98 | 89.8 |
| *ADRA2C* | F 5’ – TACTGCAACAGCTCGCTCAAC – 3’  R 5’ – CTCCGTCGGAAGAGGATGTG – 3’ | 89 | 89.9 |

Supplementary Table 6. Primers used for the estimation of bacterial densities

| **Primer** | **Sequence (5’-3’)** | **Target gene** | **T_a_** | **Reference** |
| --- | --- | --- | --- | --- |
| U2 | Froward: ACTCCTACGGGAGGCAG  Reverse: GACTACCAGGGTATCTAATCC | 16S rRNA | 62°C | Stevenson and Weimer, 2007 |
| MH | Forward:AGCAGAATATCAGCGGGGAA  Reverse:ACCAGCAATAGCGGCCAAT | leukotoxin | 60°C | This study |
| PM | Forward:TTCGGTCGGGAACTCAAAGG  Reverse:CTCTCGCTGCCCTCTGTATG | 16S rRNA | 60°C | This study |

U2 – Universal bacterial primers

MH – *M. heamolytica* primers

PM – *P. multocida* primers

T_a_ – Annealing temperature
